# Intron Retention of DDX39A Driven by SNRPD2 is a Crucial Splicing Axis for Oncogenic MYC/Spliceosome Program in Hepatocellular Carcinoma

**DOI:** 10.1002/advs.202403387

**Published:** 2024-07-17

**Authors:** Cunjie Chang, Lina Li, Ling Su, Fan Yang, Quanxiu Zha, Mengqing Sun, Lin Tao, Menglan Wang, Kangli Song, Liangyu Jiang, Haojin Gao, Yexin Liang, Chao Xu, Caiyu Yong, Minmin Wang, Jiacheng Huang, Jing Liu, Weiwei Jin, Wenyuan Lv, Heng Dong, Qian Li, Fangtian Bu, Shuanghong Yan, Haoxiang Qi, Shujuan Zhao, Yingshuang Zhu, Yu Wang, Junping Shi, Yiting Qiao, Jian Xu, Benoit Chabot, Jianxiang Chen

**Affiliations:** ^1^ School of Pharmacy and Department of Hepatology the Affiliated Hospital of Hangzhou Normal University Hangzhou Normal University Hangzhou 311121 P. R. China; ^2^ Medical Molecular Biology Laboratory Medical College Jinhua University of Vocational Technology Jinhua 321016 P.R. China; ^3^ School of Public Health Hangzhou Normal University Hangzhou 311121 P. R. China; ^4^ Laboratory of Cancer Genomics Division of Cellular and Molecular Research National Cancer Centre Singapore 169610 Singapore; ^5^ The First Affiliated Hospital Key Laboratory of Combined Multi‐Organ Transplantation Ministry of Public Health Key Laboratory of Organ Transplantation of Zhejiang Province School of Medicine Zhejiang University Hangzhou 310003 P. R. China; ^6^ Hepatobiliary and Liver transplantation Department of Hainan Digestive Disease Center The Second Affiliated Hospital of Hainan Medical University Haikou 570216 P. R. China; ^7^ Département de Microbiologie et d'Infectiologie Faculté de Médecine et des Sciences de la Santé Université de Sherbrooke Sherbrooke QC J1E 4K8 Canada; ^8^ Key Laboratory of Elemene Class Anti‐Cancer Chinese Medicines Engineering Laboratory of Development and Application of Traditional Chinese Medicines Collaborative Innovation Center of Traditional Chinese Medicines of Zhejiang Province School of Pharmacy Hangzhou Normal University Hangzhou 311121 P. R. China

**Keywords:** digitoxin, MYC signaling, RNA splicing, Sm proteins

## Abstract

RNA splicing is a dynamic molecular process in response to environmental stimuli and is strictly regulated by the spliceosome. Sm proteins, constituents of the spliceosome, are key components that mediate splicing reactions; however, their potential role in hepatocellular carcinoma (HCC) is poorly understood. In the study, SNRPD2 (PD2) is found to be the most highly upregulated Sm protein in HCC and to act as an oncogene. PD2 modulates DDX39A intron retention together with HNRNPL to sustain the DDX39A short variant (39A_S) expression. Mechanistically, 39A_S can mediate MYC mRNA nuclear export to maintain high MYC protein expression, while MYC in turn potentiates PD2 transcription. Importantly, digitoxin can directly interact with PD2 and has a notable cancer‐suppressive effect on HCC. The study reveals a novel mechanism by which DDX39A senses oncogenic MYC signaling and undergoes splicing via PD2 to form a positive feedback loop in HCC, which can be targeted by digitoxin.

## Graphical abstract

Sm proteins SNRPD2 (PD2) is highly upregulated in HCC and modulates an intron retention event on DDX39A pre‐mRNA, sustaining the expression of its short variant (39A_S). 39A_S maintains high MYC protein levels by mediating MYC mRNA nuclear export, while MYC promotes PD2 transcription, forming a positive feedback loop. Digitoxin, an FDA‐approved drug, disrupts this PD2/39A_S/MYC circuit, effectively inhibiting HCC growth.

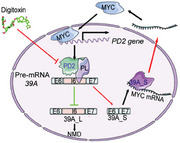

## 1 Introduction

Tumor heterogeneity has been a major hurdle for the successful treatment of cancers, including hepatocellular carcinoma (HCC).^[^ ^1^ ^]^ Alternative splicing (AS) of pre‐mRNAs is a precisely modulated posttranscriptional process in eukaryotic cells that occurs in response to environmental stimuli and contributes to RNA, protein, and cell diversity.^[^ ^2^ ^]^ Dysregulation of the AS network has been linked with carcinogenesis. Therefore, in‐depth exploration of the underlying molecular mechanisms of AS during hepatocarcinogenesis is urgently needed.

More than 90% of human protein‐coding transcripts undergo AS leading to differential inclusion or exclusion of exon and intron cassettes, which yield multiple mRNA isoforms to ensure cell‐specific spatiotemporal proteome diversity.^[^ ^3^ ^]^ This evolutionarily essential process is catalyzed by a highly dynamic multisubunit complex, the spliceosome, which consists of five small nuclear ribonucleoproteins (snRNPs; U1, U2, U4, U5, and U6), each of which is composed of one specific small nuclear RNA (snRNA) and >150 accessory proteins.^[^ ^4^ ^]^

Disruption of AS in cancer through mutation or altered expression of these accessory proteins affects cancer hallmarks. Sm proteins are core components of snRNP in spliceosome. Although the prognostic potential of Sm proteins, including SmD1 and SmE, has been reported, the expression profiles of these proteins in cancer and the detailed mechanisms underlying how they function are largely unclear.^[^ ^5^ ^]^ Through analysis of The Cancer Genome Atlas (TCGA), we found that the 7 Sm genes are differentially expressed in cancers; SNRPD2 (PD2) is the most highly expressed Sm gene, suggesting its potential oncogenic role in a Sm ring‐independent mechanism. Silencing PD2 has been linked to impaired proliferation and with G2/M cell cycle arrest in HCC cell lines.^[^ ^6^ ^]^ However, the detailed mechanism of PD2 in HCC has not been fully characterized.

MYC dysregulation occurs in >50% of tumors and is frequently associated with a poor prognosis. However, direct targeting of MYC has been proven clinically difficult due to the lack of small‐molecule binding sites. Thus, further investigations have focused on identifying potential pathways through which MYC is required to drive cancer growth. To that end, pre‐mRNA splicing has emerged as a potential vulnerability factor in MYC‐driven cancers. MYC regulates AS through modulating spliceosomal proteins.^[^ ^7^ ^]^ Conversely, MYC is also modulated by AS at the mRNA expression, protein stabilization, and transcriptional activity levels.^[^ ^8^ ^]^ Furthermore, MYC‐driven cancers are dependent on diverse spliceosome components. In HCC, MYC‐induced upregulation of SNRPB, MTR4, and hnRNPH1/2 is necessary for MYC‐driven tumorigenesis.^[^ ^9^ ^]^ These findings suggest that there are complex links between MYC and splicing machinery in cancer. However, the key sensors for MYC/splicing regulatory signaling in HCC are unclear.

In our study, we demonstrated that DDX39A short transcript 39A_S, which acts as a critical downstream splicing event, senses the integrity of Sm protein to upregulate PD2, by modulating MYC mRNA nuclear export and sustaining its expression. This PD2/39A_S/MYC circuit acts as a checkpoint to modulate the balance between highly effective transcription and splicing and maintains the proliferation of HCC cells. Moreover, an FDA‐approved drug, digitoxin, was evaluated and confirmed to directly bind to PD2 and disturb the PD2/39A_S/MYC circuit to effectively prevent HCC tumorigenesis.

## 2 Results

### 2.1 The Spliceosomal Factor PD2 is Aberrantly Upregulated in HCC, and this Phenotype is Associated with a Poor Patient Prognosis

**Figure 1:**
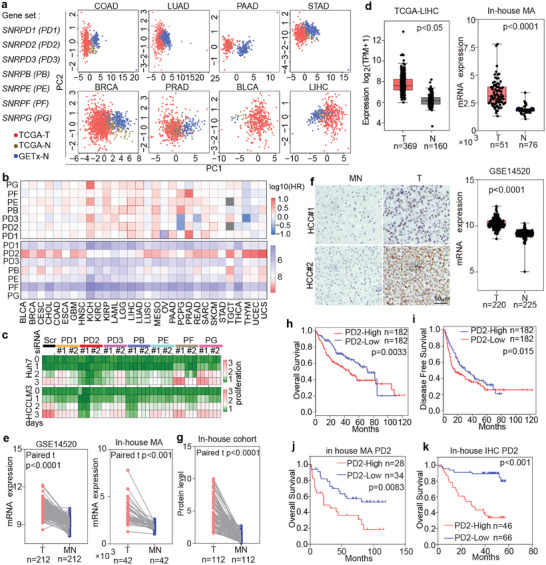
PD2 is highly expressed in HCC and negatively correlated with OS. a) PCA utilizing Sm proteins gene‐set to separate tumor from normal tissue in TCGA cohort. b) Heatmap based on hazard ratio of OS analysis by patients with high or low Sm proteins (top panel), or based on 7 Sm proteins expression across 32 cancer types (bottom panel). c) Proliferation index of tumor cells treated with siRNA against Sm proteins in Huh7 and HCCLM3 cells. d) Boxplots of PD2 expression in HCC tumor (T) and normal (N) in different datasets. Statistical analysis was performed using the Two‐tailed *t*‐test. e) PD2 mRNA expression in primary HCCs and corresponding paired tissues from GSE14520 or in‐house MA dataset. f) Representative images of PD2 IHC staining on tumor sections from an in‐house cohort. Scale bar: 50 µm. g) Semi‐quantitative analysis of PD2 IHC. h,i) OS and DFS analysis of HCC patients with PD2 high and low expression by TCGA dataset (median as the cut‐off value). j) Kaplan‐Meier analysis of PD2 mRNA levels with OS outcome in HCC patients by in‐house MA datasets. k) OS analysis on high and low PD2 expression by IHC in 112 HCC patients. Two‐tailed, paired *t*‐test is used for (e and g). ^**^ *p* < 0.01; ^***^ *p* < 0.001. Source data are provided as a Source Data file.

To assess the potential roles of spliceosomal Sm proteins in human cancer, seven well‐established Sm factors [SNRPD1 (PD1), PD2, SNRPD3 (PD3), SNRPB (PB), SNRPE (PE), SNRPF (PF) and SNRPG (PG)] were selected for investigation. Unsupervised clustering of TCGA and Genotype‐Tissue Expression (GTEx) database samples was performed via principal component analysis (PCA) based on the gene expression of Sm factors. The expression profile of Sm gene performed well in distinguishing tumor and normal samples in several types of cancers with relatively high mortality rates (**Figure** **1**a), indicating a cancer‐related role for Sm proteins. To further verify the association between the expression of Sm genes and patient prognosis, a comprehensive pancancer analysis of TCGA data comprising 30 types of cancer was performed, and revealed that the expression of Sm genes was significantly associated with the overall survival (OS) of LIHC patients (Figure 1b upper panel). Interestingly, as a reported heptamer complex, the mRNA expression of these Sm factors showed diverse patterns across cancers; PD2 was the most notably upregulated one in cancers, especially in liver‐related cancers (e.g., HCC or cholangiocarcinoma) (Figure 1b bottom panel; Figure S1a, Supporting Information).

Sm proteins are related to the prognosis of some cancers; however, the detailed roles and regulatory mechanisms of them are unclear.^[^ ^10^ ^]^ To evaluate the key Sm protein in HCC, we silenced their expression in Huh7, HCCLM3, SMMC7721, or SNU449 cells (Figure S1b, Supporting Information), and the results showed that silencing *PD2* expression markedly inhibited the growth of cells, while the effect was less pronounced when other *Sm* genes were silenced (Figure 1c; Figure S1c, Supporting Information).

To further verify PD2 expression in HCC tissues, HCC‐related gene expression databases were analyzed, and experimental validation was performed with independent cohorts. Highly upregulated *PD2* expression in HCC was observed in three previously established independent cohorts (Figure 1d; Table S1, Supporting Information).^[^ ^11^ ^]^ Moreover, according to analysis of data on 212 or 42 paired tissues from GSE14520 or in‐house MA (E‐MEXP‐84 and E‐TABM‐292) datasets, *PD2* expression was significantly elevated in tumor (T) compared to matched normal (MN) tissues (Figure 1e). PD2 protein expression was further evaluated by immunohistochemistry (IHC) staining of 112 pairs of HCC samples (Table S2, Supporting Information), which revealed that PD2 was localized mainly in nucleus and was markedly upregulated in HCC T (Figure 1f,g). Furthermore, survival analysis was performed for patients grouped based on *PD2* gene or protein expression. The results indicated that patients with high PD2 gene or protein expression had significantly worse OS or disease‐free survival (DFS) than those with low PD2 expression (Figure 1h–k).

### 2.2 MYC Signaling is a Key Driver that Sustains High PD2 Expression in HCC

**Figure 2:**
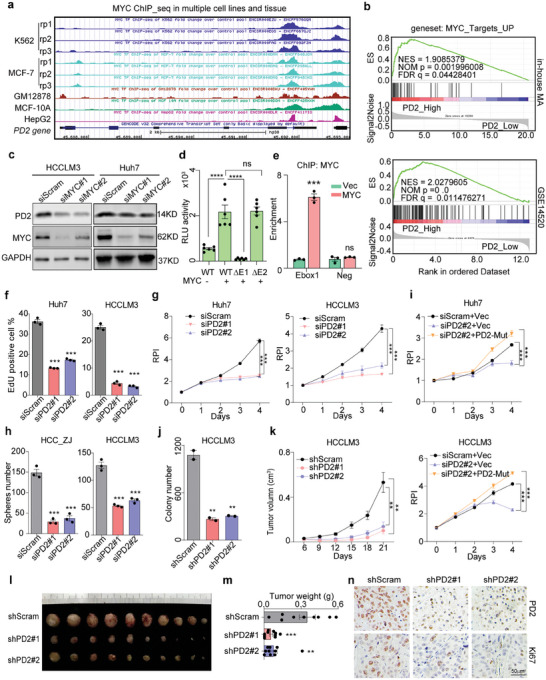
MYC directly promotes PD2 transcription and PD2 is crucial for HCC progression. a) Schematic of human PD2 locus on chromosome 19 (UCSC genome version hg38). ChIP‐seq signals of MYC in K562, MCF‐7, GM12878, MCF‐10A and HepG2 (data from ENCODE). b) GSEA of different expressed genes in PD2‐high compared to PD2‐low group from GSE14520 and in‐house MA datasets. c) The protein level of PD2 was detected by WB upon MYC knockdown. d) Ebox1 or Ebox2 was deleted form PD2 luciferase reporter plasmid to detect luciferase activity of cell co‐transfected with MYC‐OE/Vec, respectively. (n = 6, data are shown as the mean ± SD). e) ChIP analysis of HEK293T transfected with a control or MYC‐OE plasmid. Binding of MYC at the promoter was quantified by qPCR, and is shown as the relative enrichment compared with IgG. Results are the means ± SEM by three independent experiments. f) Quantitative analysis of 5‐ethynyl‐20‐deoxyuridine (EdU) staining. g) CCK‐8 and in control and PD2 knockdown cells. h) Expression of a rescue construct partially restores cell proliferation. i) Quantitative analysis of spheres number in control and PD2 knockdown cells. j) Quantitative analysis of Colony formation in soft agar for stable PD2 knockdown. k) Morphologies of collected tumors. l) Tumor weights were measured (n = 10). m) Mouse xenograft growth curves in shScram and shPD2 cells. n) IHC for Ki67 and PD2 in xenograft. Scale bars: 50 µm. Data were presented as mean ± SEM, unpaired *t*‐test is used for (d, e, f, i, j, l), two way ANOVA is used for (g, h, m);^*^ *p* < 0.05; ^**^ *p* < 0.01; ^***^ *p* < 0.001; ns indicates non‐significant.

To clarify the mechanism underlying the maintenance of high PD2 expression mentioned above, the *PD2* promoter elements were analyzed for potential transcriptional binding factors, which revealed two notable MYC‐binding elements, named *E‐boxes*, located at ‐115 or ‐10 bp (Figure S2a, Supporting Information). Recently, a regulatory link between MYC and spliceosomal proteins has been indicated for lymphomagenesis and breast cancer; thus, we aimed to perform additional assays on MYC‐related regulation in HCC.^[^ ^12^ ^]^ First, an investigation of UCSC ChIP‐seq data with an anti‐MYC antibody revealed two MYC‐binding peaks on *PD2* promoter in a hepatoma cell line HepG2 and other cell lines (**Figure** **2**a). Then, gene set enrichment analysis (GSEA) was performed using the HCC data, which revealed that the MYC‐target‐UP gene set was significantly enriched in the samples with high *PD2* expression (Figure 2b).

To assess whether MYC could directly regulate PD2 expression, we silenced MYC expression in HCC cells. PCR and western blotting (WB) assays indicated that MYC knockdown could significantly suppress PD2 expression (Figure S2c, Supporting Information; Figure 2c). Moreover, forced expression of MYC transiently upregulated PD2 protein expression in a dose‐dependent manner (Figure S2b, Supporting Information). To further confirm the transcriptional regulation of *PD2* by MYC, three *PD2* promoter constructs (‐1000 to ‐1 bp) were cloned into the pGL3‐luciferase reporter plasmid, named *PD2*‐luci (containing the wild‐type *E‐box1* and *E‐box2* elements) or ∆E1‐luci or ∆E2‐luci (containing either *E‐box1* or *2* deletions, respectively) (Figure S2a, Supporting Information). MYC overexpression resulted in significant dose‐dependent activation of *PD2*‐luci construct (Figure S2d, Supporting Information) and also significantly increased the luciferase activity of ∆E2‐luci but not ∆E1‐luci (Figure 2d). Furthermore, a chromatin immunoprecipitation (ChIP) assay confirmed that MYC could bind to *E‐box1* but not the negative control region (Figure 2e; Figure S2e, Supporting Information).

### 2.3 PD2 is Essential for HCC Progression

To assess the role of PD2 in HCC, two HCC cell lines with high *PD2* expression, Huh7 and HCCLM3, were selected for *PD2* knockdown (Figure S3a,b, Supporting Information). PD2 knockdown markedly suppressed the proliferation (Figure 2f; Figure S3i, Supporting Information) and decreased the viability (Figure 2g) of HCC cells. To exclude the off‐target effect of siRNA, we generated a siRNA‐resistant *PD2* expression clone named PD2‐Mut (Figure S3c, Supporting Information). Transfection of PD2‐Mut into PD2‐silenced cells attenuated the inhibitory effect of *PD2* knockdown on cell viability (Figure 2h; Figure S3d, Supporting Information), which indicates that PD2 expression directly potentiates the growth potential of HCC cells.

Furthermore, we found that the genes enriched in samples with high *PD2* expression were significantly associated with stemness and invasion through GSEA analysis (Figure S3e,f, Supporting Information). To verify the stemness, a patient‐derived primary HCC cell line named HCC_ZJ was generated, and the oncosphere assay was performed to evaluate the cancer stem cell‐like properties.^[^ ^13^ ^]^ Moreover, the transwell invasion assay was used to evaluate the invasiveness of HCC cells. The results showed that the cancer stem cell‐like properties and invasive abilities of HCC cells were markedly attenuated when endogenous PD2 was depleted (Figure 2i; Figure S3g,h, Supporting Information), indicating that PD2 plays a key role in sustaining the stemness and invasiveness of HCC cells.

To evaluate the oncogenicity of HCC cells, an anchorage‐independent colony formation assay by soft agar was employed to show that stable knockdown of PD2 inhibited colony formation (Figure 2j; Figure S3j,k, Supporting Information). To further assess the tumourigenesis of PD2 in vivo, a subcutaneous HCCLM3 xenograft mouse model was established by injecting mice with HCCLM3 with stable PD2 or Scram knockdown; the results indicated that PD2 knockdown xenografts were smaller in terms of volume (Figure 2k) and weight (Figure 2l) and grew slower (Figure 2m) than control xenografts. Moreover, IHC staining of the xenografts indicated that PD2 expression was stably silenced and associated with a reduction in the proportion of Ki67‐positive cells (Figure 2n).

### 2.4 PD2 Expression is Closely Associated with *DDX39A* Splicing and Promotes Expression of the Short Variant 39A_S in HCC

**Figure 3:**
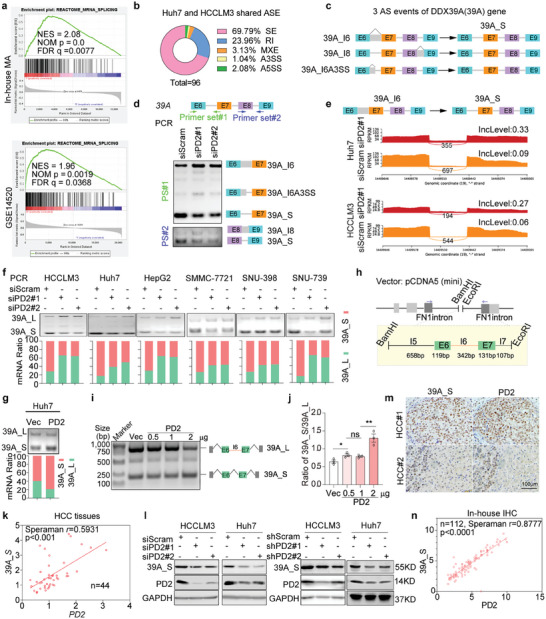
PD2 regulates DDX39A splicing. a) GSEA of different mRNA splicing related genes in PD2‐high compared to PD2‐low group from GSE14520 or in‐house MA dataset. b) The AS events regulated by PD2. c) Diagram of the splicing variants of DDX39A mRNA. d) Top: diagram of the primers for PCR detection of intron 6 (primer set # 1) and intron 8 (primer set # 2). Bottom: DDX39A isoforms were examined by RT‐PCR in HCCLM3. e) Reads count of the spliced DDX39A variants by RNA_Seq of cells as indicated. f) Validation of the expression of 39A_I6 (39A_L) and 39A_S isoforms by PCR in PD2‐slienced HCC cell lines. mRNA ratio of 39A_S and 39A_L was determined by Image J. g) Expression of 39A_L and 39A_S isoforms was detected by PCR. h) The construction of *39A* pre‐mRNA minigene. i) Validation of the expression of 39A_L and 39A_S isoforms by PCR in Hek293T cells. j) mRNA ratio of 39A_S and 39A_L was determined by Image J. k) Positive correlation between 39A_S and PD2 in mRNA level in 44 HCC and MN tissues. l) WB analysis of 39A_S protein in cells. m) Typical IHC staining of PD2 and 39A_S in outgrowing tumor slices from a tissue array containing 112 HCC tissues. Scale bars: 100 µm. n) Positive correlation between 39A_S and PD2 by quantifying the intensity of IHC staining. Pearson's correlation analysis was performed to (h,k).^*^ *p* < 0.05; ^**^ *p* < 0.01; ^***^ *p* < 0.001. ns indicates non‐significant.

To dissect the mechanism of PD2 in HCC, we performed GSEA with GSE14520 and in‐house MA data. The results indicated that the genes significantly enriched in PD2‐highly‐expressed samples were associated mainly with mRNA splicing (**Figure** 3a), indicating an association between PD2 and splicing regulation in HCC. Next, we performed RNA sequencing (RNA‐seq) to identify PD2‐linked AS events (ASEs) between siScram‐ or siPD2‐treated HCC cells. Replicate multivariate analysis of transcript splicing (rMATS) was used to compute and quantify the differentially enriched ASEs associated with PD2 which were classified into five categories (Figure S4a, Supporting Information).^[^ ^14^ ^]^ A total of 5,421 or 884 PD2‐linked ASEs were observed (Figure S4b, Supporting Information) and indicated the dual role of PD2 as a splicing activator (repressor) in inducing intron exclusion (activation) or exon inclusion (repression) (Figure S4c, Supporting Information). Among all ASEs, 96 significantly differentially enriched ASEs were observed, most of which were SE or RI events (Figure 3b). To verify the AS profile, we selected the representative ASEs for two cell lines (p < 0.05, FDR < 0.05) and validated the results independent PCR (Figure S4d, Supporting Information).

To characterize PD2‐linked HCC‐associated ASEs (PHAs), the screened ASEs were further compared with an in‐house AS dataset generated from 20 pairs of HBV‐related HCC samples (Figure S4e,f and Table S3, Supporting Information). Interestingly, three PHAs observed in all samples were ASEs of one gene – *DDX39A*; the splicing patterns of them were different, but these events ultimately generated the same mature mRNA, 39A_S (Figure 3c). The human *DDX39A* gene contains 11 exons, and additional AS isoforms have been described previously.^[^ ^15^ ^]^ Here, four isoforms of *DDX39A* were detected in Huh7 cells by PCR (Figure 3d). The expression of three isoforms, generated by the retention of intron 6 or 8 (named 39A_I6 or 39A_I8) or the A3SS of intron 6 (named 39A_I6A3SS), significantly increased when PD2 knockdown, while the short isoform 39A_S expression markedly decreased (Figure 3d). As 39A_I6 and 39A_S were highly expressed in HCC cells (Figure 3d) and 39A_S was the only isoform encoding the DDX39A protein, for the following experiments, we focused only on investigating long isoform 39A_I6 (named 39A_L) and the short isoform 39A_S.

To confirm the regulatory effect of PD2 on 39A_L and 39A_S, RNA‐seq data were analyzed that *PD2* knockdown could potentiate DDX39A intron 6 retention (Figure 3e; Figure S4g, Supporting Information). *PD2* was subsequently knocked down in 6 HCC cell lines, which potentiated the expression of the 39A_L transcript but markedly attenuated the expression of 39A_S transcript, leading to aberrant changes in the percent spliced‐in (PSI) (Figure 3f; Figure S4h,Supporting Information). Conversely, PD2 overexpression inhibited 39A_L transcript expression but markedly promoted 39A_S transcript expression (Figure 3g). To further confirm the splicing regulation of 39A by PD2, we generated a minigene reporter by inserting a DNA fragment including intron 5, exon 5, intron 6, exon 6, and intron 7 into pCDNA5 plasmid (Figure 3h). Upon PD2 overexpression, the short splicing fragment of 39A without intron 6 was increased by overexpressed PD2 in a dose‐dependent manner (Figure 3i,j). The 39A_S transcript is the only established isoform that produces the DDX39A protein, and 39A_L transcripts with a typical premature termination codon (PTC) between exons 6 and 7 were predicted to be a target of the nonsense‐mediated mRNA decay (NMD) pathway (Figure S4i, Supporting Information). This suggested that 39A_L might act as a noncoding transcript and might be degraded via the endogenous NMD pathway. For further confirmation, upstream frameshift 1 (UPF1), a core upstream activator of the NMD pathway, was depleted in Huh7 (Figure S4j, Supporting Information); this restored the detectable expression of 39A_L transcript to a very low level (Figure S4k, Supporting Information), confirming that 39A_L could be degraded via the UPF1/NMD pathway. Moreover, in a set of 22 paired HCC tissues, PD2 gene expression was confirmed to be strongly correlated with 39A_S expression (Figure 3k).

To verify the regulatory effect of PD2/39A_S signaling at the protein level, we constructed transient and stable knockdown of PD2 cells which revealed an attenuated protein expression of 39A_S (Figure 3l). However, the import of PD2‐Mut could reverse the expression of 39A_S (Figure S4l,m, Supporting Information). Moreover, forced expression of PD2 upregulated the protein expression of 39A_S (Figure S4n, Supporting Information). To assess the co‐expression of PD2 with 39A_S in HCC tissues, sequential HCC slides were subjected to PD2 or 39A_S expression and colocalization analysis, and the results showed that PD2 expression was positively correlated with 39A_S expression in the same regions of the tissues (Figure 3m). In an analysis of IHC results for 112 HCC patient samples (Table S2, Supporting Information), PD2 expression was found to be significantly correlated with 39A_S expression (Figure 3n).

### 2.5 39A_S is a Crucial Downstream Effector that Mediates the Tumorigenic Effects of PD2 in HCC

**Figure 4:**
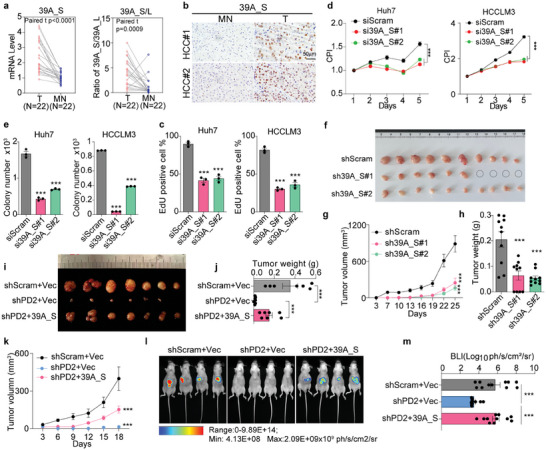
39A_S mediate the oncogenic function of PD2. a) Normalized 39A_S expression and 39A_S/39A_L expression ratios in 22 pairs of HCC. b) Representative IHC staining images of high or low 39A_S expression in HCC tissues, scale bar: 50 µm. c) EdU proliferation, d) CCK8 or e) 2D‐colony formation was analyzed in HCC cells transfected with siScram or si39A_S (n = 3). f–h) Mouse tumorigenic assays of HCCLM3 with control or 39A_S stably knockdown (n = 10). g) Tumor growth, f) tumor images, and h) tumor weight were shown. i–k) The representative tumor pictures (i), tumor volume (j), and tumor growth (k) of the mouse subcutaneous tumorigenic assay of shScram+Vec, shPD2+Vec, and shPD2+39A_S HCCLM3 cells (n = 7). l) The hepatic tumors were monitored by Bioluminescence imaging (BLI). m) Normalized photon flux for the indicated time is presented (n = 11). Mean ± SEM, two‐tailed, unpaired *t*‐test is used for (e, c, j, h, m), two‐way anova is used for (d, g); ^*^ *p* < 0.05; ^**^ *p* < 0.01; ^***^ *p* < 0.001.

To reveal the potential role of 39A_S in HCC, we first evaluated RNA expression of two isoforms in HCC tissues. We found that the expression of 39A_S and the ratio of 39A_S to 39A_L were significantly increased in HCC T compared to MN tissues, while 39A_L expression was significantly decreased (**Figure** **4**a; Figure S5a, Supporting Information). Consistently, 39A_S protein expression, detected via IHC staining, was also markedly greater in T than MN tissues (Figure 4b), indicating the potential hepatocarcinogenic role of 39A_S.

The 39A_L isoform was previously confirmed to be a noncoding RNA degraded via the endogenous NMD pathway. To assess the role of 39A_L and 39A_S in HCC, we designed siRNAs specific for 39A_L or 39A_S (Figure S5b, Supporting Information). Given that the endogenous expression of 39A_L is very low in parental cells, siRNAs targeting 39A_L and UPF1 were co‐transfected into HCCLM3 cells (Figure S5c, Supporting Information). The results showed that knockdown of 39A_L had no significant effect on cell proliferation (Figure S5d, Supporting Information). We further silenced 39A_S expression but not 39A_L within the cells (Figure S5e, Supporting Information). Inhibition of 39A_S expression significantly suppressed the proliferation (Figure 4c; Figure S5f, Supporting Information), growth (Figure 4d), colony formation (Figure 4e), stemness (Figure S5g, Supporting Information) and migration (Figure S5h, Supporting Information) of HCC cells. Furthermore, we generated HCCLM3 cells with stable silencing of 39A_S via shRNA transfection (Figure S5i, Supporting Information) and the results showed that 39A_S silencing led to a marked decrease in tumor burden and growth rate (Figure 4f–h).

To further evaluate whether 39A_S mediates the oncogenic effect of PD2 in HCC, the 39A_S clone was transfected and stably re‐expressed into PD2‐silenced cells (Figure S5j, Supporting Information), and cell viability was partially rescued by 39A_S (Figure S5k, Supporting Information). To further confirm this finding, we reintroduced 39A_S into stable PD2‐knockdown HCCLM3‐luciferase cells (Figure S5l, Supporting Information), which were subsequently subcutaneously injected into nude mice. We found that ectopic re‐expression of 39A_S in these cells partially restored xenograft tumor growth suppressed due to PD2 deletion (Figure 4i–k). Additionally, we used an orthotopic transplantation model in nude mice. The luciferase activity in the liver was detected to monitor tumor growth in vivo. Significant restoration of tumor growth was observed when 39A_S was re‐expressed in stable PD2‐knockdown cells (Figure 4l,m).

### 2.6 PD2 Primes intron 6 Excluding *DDX39A* Mainly by Recruiting the Splicing Factor HNRNPL

**Figure 5:**
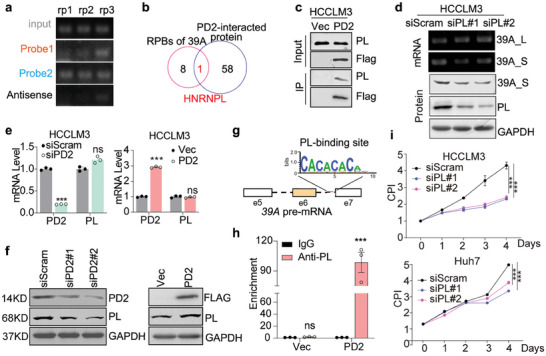
PD2 recruits HNRNPL to enhance DDX39A splicing. a) Biotinylated probes were incubated with nuclear extracts and collected with streptavidin beads. Isolated RNA was detected by PCR; Isolated proteins were subjected to MS. b) The common RPB proteins binding with both DDX39A mRNA constructed and PD2. c) IP validation of the interaction of PD2 and HNRNPL. d) The mRNA level of two spliced variants and 39A_S proteins level was detected upon HNRNPL knockdown. e) mRNA or f) protein level of HNRNPL and PD2 in HCCLM3 treated with siScram or siPD2 (n = 3). g) PL‐binding motif was shown in *DDX39A* pre‐mRNA. h) RIP assay detected the HNRNPL binding at the *DDX39A* pre‐mRNA in PD2 overexpression and control cells (n = 3). i) The cell growth were analyzed in HCCLM3 and Huh7 cells transfected with siPL. Mean ± SD, two‐tailed, unpaired *t*‐test is used for (e,h), two way ANOVA is used for (i); ^**^ *p* < 0.01; ^***^ *p* < 0.001; ns indicates non‐significant.

To identify potential AS factors that directly bind and regulate *DDX39A* pre‐mRNAs, RNA pulldown coupled with mass spectrometry (MS) was performed. Two sense probes and one antisense biotin‐labeled probe located in intron 6 were designed and synthesized (Figure S6a, Supporting Information). An RNA pulldown assay suggested that biotin‐labeled probe 1 and 2 but not the antisense control probe could successfully pull down the RNA (**Figure** **5**a). MS analysis further revealed 9 proteins excluding PD2 that potentially bind to *DDX39A* pre‐mRNA (Figure S6b, Supporting Information), which suggested that PD2 may not directly bind to *DDX39A* pre‐mRNA. Thus, To identify a splice factor that not only directly binds to 39A pre‐mRNA but also interacts with PD2, PD2‐interacting proteins were explored via IP‒MS and further compared with *DDX39A* pre‐mRNA‐binding proteins; the results revealed that the splicing factor HNRNPL (PL) is a candidate protein through which PD2 potentially modulates *DDX39A* splicing (Figure 5b).

We further confirmed the potential interaction between PD2 and PL via an independent IP assay in HCCLM3 cells (Figure 5c, Supporting Information). Silencing of PL restored 39A_L expression and reduced the mRNA and protein levels of 39A_S (Figure 5d), indicating that PL could regulate DDX39A splicing in a manner similar to that of PD2. However, PD2 expression interruption had no significant effect on PL RNA or protein expression (Figure 5e,f, Supporting Information).

To verify whether PL could directly bind to the intron 6, sequence analysis was performed, and a conserved PL‐binding site was found in intron 6 of *DDX39A* pre‐mRNA (Figure 5g). RIP further confirmed that PL antibody could markedly pull‐down RNA sequences containing PL‐binding motif in intron 6 only when PD2 was expressed (Figure 5h). To investigate whether PL plays a similar role as PD2 in HCC cells, we further performed several functional assays in HCCLM3 and Huh7. As expected, we found that PL is a potential oncogenic driver in HCCLM3 and Huh7 (Figure 5i; Figure S6c,d, Supporting Information).

### 2.7 39A_S Sustains MYC Expression by Promoting the Nuclear Export of MYC mRNA in HCC Cells

**Figure 6:**
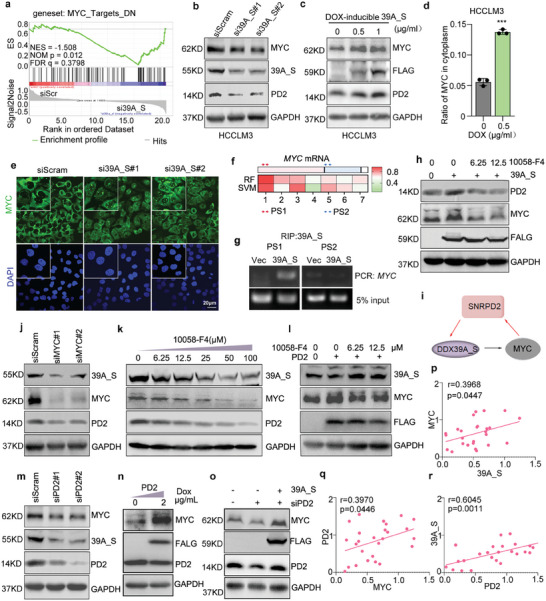
MYC/PD2/39A_S feedback circuits in HCC. a) MYC_target_up signature was enriched in si39A_S group compared to siScram group by GSEA. b) WB of PD2 and MYC proteins in HCCLM3 transfected with si39A_S or siScram. c) HCCLM3 treated with DOX in dose‐dependent manner to induce 39A_S protein expression was detected by WB. d) qPCR assay was performed to test MYC mRNA level in cytoplasm of cells treated with 0.5 µg ml^−1^ DOX. e) FISH analysis was conducted in cells treated with si39A_S or siScram using biotinylated Probe against MYC mRNA. f) Binding site of DDX39A on MYC mRNA was predicated online (http://pridb.gdcb.iastate.edu/RPISeq/). Color bar indicated the 39A_S binding affinity. PS1: primer set 1; PS2: primer set 2. PS1 and PS2 are designed for detection of MYC pulldown by DDX39A protein. g) The binding between DDX39A and MYC RNA was verified by RIP assay with anti‐DDX39A antibody, and co‐precipitated transcripts were determined using RT‐PCR. GAPDH is used as input control. h) PD2 protein level in 39A_S‐OE HCCLM3 cells treated with 10058‐F4 was examined by WB. i) The feedback loop composed with PD2, 39A_S and MYC. j,m) PD2 and 39A_S protein is examined by WB in HCCLM3 transfected with indicated siRNA. k) WB showed PD2 and 39A_S protein inhibited by treatment with 10058‐F4 in a dose‐dependent manner. l) PD2, 39A_S and MYC protein in PD2‐OE cells treated with MYC inhibitor was determined by WB. n) HCCLM3 was infected with dox‐inducible (2 µg ml^−1^) PD2 expression lentivirus, and PD2, 39A_S, and MYC protein was determined by WB. o) PD2, 39A_S and MYC protein was determined by WB in PD2 knockdown cells treated with or without dox‐inducible 39A_S expression. p–r) PD2, 39A_S, and MYC proteins were detected by WB in 13 pairs of HCC tissues. Intensity of WB was quantified by Image J. Correlation of the expression of MYC and 39A_S, PD2 and MYC, or PD2 and 39A_S, was shown in (p), (q), or (r). Pearson's correlation analysis was performed to (p,q,r).

To explore the mechanism by which 39A_S exerts its effect, RNA‐Seq was performed using 39A_S‐silenced and control cells. Interestingly, GSEA revealed that the MYC_target_DN gene set was significantly enriched in 39A_S‐silenced cells (**Figure** **6**a), which indicates the tight positive association of 39A_S with MYC in HCC cells. The decrease in MYC expression after 39A_S knockdown notably attracted our attention (Figure 6b). In contrast, the MYC protein level increased in a dose‐dependent manner when 39A_S expression was gradually increased (Figure 6c). In order to exclude the influence of doxycycline on MYC/PD2/39A expression, the control cells infected with lentivirus expressing empty vector were also treated with doxycycline (Figure S7a, Supporting Information). DDX39A was previously reported to be a critical modulator of mRNA export from the nucleus.^[^ ^16^ ^]^ Thus, to track subcellular distribution of MYC mRNA, we fractionated nuclear and cytoplasmic mRNA and evaluated the expression of *MYC* or *U6* in fractions (Figure S7b,c, Supporting Information). The results revealed that 39A_S overexpression promoted MYC mRNA enrichment in cytoplasm (Figure 6d), and this result was further validated by fluorescence in situ hybridization (FISH) using a MYC‐specific probe (Figure 6e; Figure S7d, Supporting Information). To assess whether MYC mRNA in the cytoplasm could be translated into a protein, the half‐life of MYC protein in 39A_S‐overexpressing cells and control‐overexpressing cells was detected via MG132 treatment, and the results showed that 39A_S sustains MYC protein expression (Figure S7e,f, Supporting Information).

To determine whether 39A_S could directly bind to MYC mRNA, RPISeq was used to predict the probability of the interaction.^[^ ^17^ ^]^ Region 1 or 5 had stronger or weaker binding affinities, respectively (Figure 6f). Primers localized in region 1 or 5 were applied to perform RIP assay for validation (Figure 6f). The observation that 39A_S was enriched mainly in region 1 indicated that 39A_S directly bound to region 1 (Figure 6g) and promoted the translocation of MYC mRNA to cytoplasm to enhance MYC expression.

Our results have shown that 39A_S regulated PD2 protein expression reversely via promoting MYC expression (Figure 6b‐c), and this effect could be inhibited by the MYC inhibitor 10058‐F4 (Figure 6h). Together with Figures 2 and 3, our findings suggest the presence of a positive feedback loop among MYC, PD2 and 39A_S (Figure 6i). To further confirm it, siRNA or 10058‐F4 was performed to inhibit MYC expression, and 39A_S and PD2 protein expression were reduced (Figure 6j,k). MYC inhibition can not reverse 39A_S expression induced by PD2 overexpression (Figure 6l), indicating MYC did not mediate the regulation of 39A by PD2. Furthermore, PD2 regulated MYC expression in a reverse manner (Figure 6m,n), and this effect was mediated by 39A_S (Figure 6o). The expression of MYC, PD2, and 39A_S by WB were positively correlated with each other in a cohort of 13 pairs of HCC tissues, which confirmed the feedback loop in HCC (Figure S7g, Supporting Information; Figure 6p–r).

### 2.8 Digitoxin acts as a Novel Inhibitor of PD2 to Suppress PD2/39A_S/MYC Signaling Circuit and Corresponding Functions

**Figure 7:**
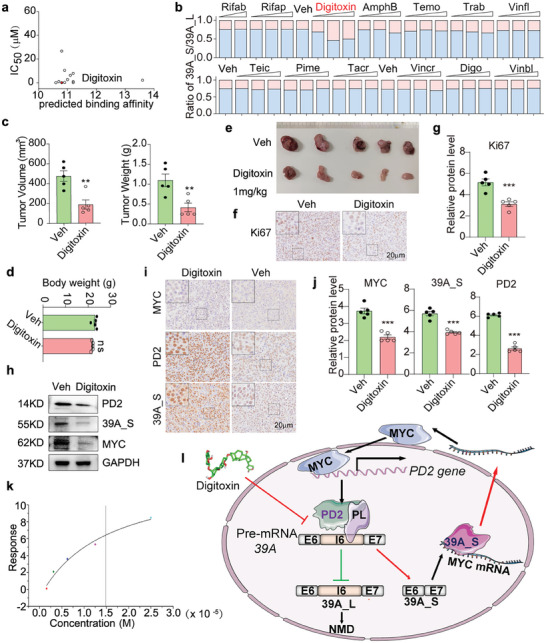
Digitoxin inhibits MYC/PD2/39A feedback circuits in HCC. a) The predicted binding affinity and IC_50_ of top 13 drugs through macromolecular docking. b) PSI of 39A_S and 39A_L in cells treated with 13 selected drugs. Veh: Vehicle, Rifab: Rifabutin, Rifap: Rifapentine, AmphB: Amphotericin B, Temo: Temoporfin, Trab: Trabectedin, Vinfl: Vinflunine, Teic: Teicoplanin, Pime: Pimecrolimus, Tacr: Tecrolimus, Vincr: Vincristine, Digo: Digoxin, Vinbl: Vinblastine. c–e) Hepa1‐6 cell was injected subcutaneously in C57BL/6J. The mice were treated with intraperitoneal injection of digitoxin. c) Tumor weight and tumor volume, d) body weight, e) tumor image were shown (n = 5). f) Representative IHC staining images of Ki67 expression, scale bar: 20 µm. g) Semi‐quantitative analysis of Ki67 IHC. h) Total protein was harvested from tumor tissue for WB analysis l of PD2, 39A_S, and MYC. i) Representative IHC staining images of 39A_S, PD2, and MYC expression, scale bar: 20 µm. j) Semi‐quantitative analysis of 39A_S, PD2 and MYC IHC. k) The binding of PD2 with digitoxin was determined via SPR. l) Working model of MYC/PD2/39A_S feedback loop. two‐tailed, unpaired *t*‐test is used for (c,d).

To find PD2 inhibitors, we acquired the established cocrystal structure of PD2 with PD1 from UniProt for screening.^[^ ^18^ ^]^ Molecular docking was subsequently utilized for screening in FDA‐approved drugs that potentially bind to the key pocket with the intention of disrupting the AS function of PD2 from Therapeutic Target Database (https://db.idrblab.net/ttd/). We ultimately selected top listed 13 drugs based on predicated binding affinity and IC_50_ values in HCCLM3 cells (**Figure** **7**a); 3D docking model of PD2 with digitoxin was shown in Figure S8a (Supporting Information). Subsequently, PCR was employed to determine the relative ratio of 39A_L and 39A_S variants in cells treated with these drugs. Significantly, the incubation with digitoxin notably induced dose‐dependent upregulation of 39A_L expression, along with substantial inhibition of 39A_S expression and cell growth (Figure S8b, Supporting Information; Figure 7b).

Digitoxin stands out among the cardiac glycosides that have undergone comprehensive studies and has a firmly established clinical profile and have been proven to have pronounced anticancer efficacy.^[^ ^19^ ^]^ Here, we found that digitoxin significantly inhibited HCC growth and cell proliferation but had no effect on body weight (Figure 7c–g). Additionally, it markedly suppressed the expression of PD2, MYC, and 39A_S (Figure 7h–j), indicating its inhibitory role in PD2/39A_S/MYC circuit.

To confirm the affinity of digitoxin for PD2, we expressed and purified PD2 protein in vitro (Figure S8c, Supporting Information) and conducted a surface plasmon resonance (SPR) assay (Figure 7k; Figure S8d, Supporting Information). The results demonstrated that PD2 captured on Chip CM5 could bind to digitoxin specifically with an affinity constant of 14.9 µm (Figure 7k; Figure S8d, Supporting Information). Thus, for the first time, we confirmed that digitoxin is an inhibitor for PD2 that targets the PD2/39A_S/MYC feedback loop in HCC.

In summary, we identified a novel molecular circuit composed of PD2/39A_S/MYC (Figure 7l) that sustains the integrity of core Sm proteins and mediates the MYC‐splicing regulatory program in the context of hepatocarcinogenesis, which can be targeted by a clinic drug – digitoxin.

## 3 Discussion

In eukaryotes, a ring of seven different but specific Sm proteins is thought to bind to small nuclear RNA at a poly (U) sequence to form the core of each snRNP complex.^[^ ^20^ ^]^ In yeast, PE and PF can form stable homo‐oligomeric ring structures in solution.^[^ ^21^ ^]^ This finding suggested that an additional working model of the Sm protein may exist in different models, especially during cancer development. In our study, PD2 was proven to promote the exclusion of intron 6 by recruiting HNRNPL, which illustrates a novel working model of PD2 in hepatocarcinogenesis. Additionally, previous studies have shown that the conserved seven‐nucleotide Sm‐binding sequence (5′‐AUUU/CUUG‐3′) is found in many RNAs.^[^ ^22^ ^]^ However, the current study has no evidence indicating direct binding of PD2 to pre‐mRNAs. Additional investigations on the roles of Sm proteins in cancer should be performed to verify this speculation and reveal the underlying mechanisms.

Targeting the spliceosomal core machinery in cancer is currently thought to be an attractive therapeutic strategy.^[^ ^23^ ^]^ However, most agents targeting spliceosomes always cause tumor off‐target effects. Previous studies have posited that MYC‐driven tumors are more sensitive to spliceosome‐targeted therapies (STTs). However, the mechanisms by which STTs selectively kill cancer cells have not been elucidated, although one mechanism might be that mis‐spliced RNA itself is a molecular trigger for tumor killing through viral mimicry.^[^ ^24^ ^]^ Other potential theories have also been proposed: for example, it has been proposed that MYC overexpression forces cells to produce high levels of constitutive splicing machinery to process highly amplified RNA and sustain splicing fidelity in cancer cells.^[^ ^12a^ ^]^ In our study, HNRNPL activated intron 6 splicing through binding to the CA repeat downstream of intron 6, which was consistent with previous findings.^[^ ^25^ ^]^

Digitoxin is a well‐known cardiac glycoside that has potential as an anticancer drug at therapeutic concentrations ranging from 0.01–10 µm.^[^ ^26^ ^]^ Epidemiological data suggest that patients who receive treatment with the cardiac glycosides digitoxin or digitoxin have a higher survival rate and lower recurrence of different cancer malignancies.^[^ ^27^ ^]^ Cancer cells seem to be more sensitive to digitoxin than normal cells are, but the reason for this difference is unknown. One study revealed that digitoxin inhibits the interaction of NFAT1 with the proximal MYC promoter to suppress MYC transcription.^[^ ^28^ ^]^ Another study reported that bufanolides have similar effects as digitoxin and play an antiproliferative role via MYC suppression.^[^ ^29^ ^]^ Our study confirmed that targeting feedback circuits is a possible mechanism by which digitoxin acts as an anticancer agent with selectivity for cancers driven by MYC signaling.

## 4 Conclusion

We therefore concluded that the MYC/39A_S/PD2 feedback circuit is essential for hepatocarcinogenesis. Disturbing any part of the circuits should prevent HCC growth. Digitoxin, a drug targeting this circuit, might be a promising therapeutic agent for precisely targeting cancer cells affected by MYC.

## 5 Experimental Section

### siRNA

The siRNAs targeting genes were purchased from Gene Pharma and transfected with Lipofectamine RNAiMAX (Thermo Fisher Scientific, 13778150). The sequences are listed in Table S6 (Supporting Information).

### Subcutaneous Xenograft Assay in Nude Mice

Male BALB/C athymic nude mice at 5–7 weeks of age were purchased from the animal center of the Cancer Institute of Chinese Academy of Medical Science (Shanghai, China). The cultures of stable HCCLM3 cells were transplanted subcutaneously implanted into the left flanks (5 × 10^6^ cells per flank) of mice. The length and width of tumors were recorded every day. Tumor volume was calculated according to the formula volume = 0.5 × length × width^2^. Once the xenograft volume exceeded 2000 mm^3^, as allowed by IACUC of Hangzhou Normal University, the mice should be euthanized.

### Statistics Analysis

To assess the statistical significance of disparities between two groups, unpaired Student's *t*‐tests were employed to compute two‐tailed *p*‐values, while Chi‐square or Fisher's exact test was also used in selected cases. The experimental outcomes were presented as mean ± SD, depending on the analysis's requirements. For the CCK8 assay, two‐way ANOVA was applied for in‐depth analysis. To determine the association between two variables, Pearson's correlation analysis was conducted. Kaplan–Meier analyses, and Cox's proportional hazards regression model. The statistical significance threshold was set at *p* < 0.05. The level of statistical significance was denoted in figures using asterisks: ^*^ for *p* < 0.05, ^**^ for *p* < 0.01, ^***^ for *p* < 0.001, and ^****^ for *p* < 0.0001. Additionally, ImageJ (NIH, version 1.8.0) was utilized to analyze the mean fluorescence density. All statistical analyses were conducted using GraphPad Prism (version 8.0). Additional details of the Materials and Methods can be found in the Supplementary Material.

## Conflict of Interest

The authors declare no conflict of interest.

## Supporting information

Supporting Information

## Data Availability

The data that support the findings of this study are available on request from the corresponding author. The data are not publicly available due to privacy or ethical restrictions.
