# Supplementary material for: Intron Retention of DDX39A Driven by SNRPD2 is a Crucial Splicing Axis for Oncogenic MYC/Spliceosome Program in Hepatocellular Carcinoma
Source: Adv Sci (Weinh). 2024 Jul 17;11(35):2403387. doi: 10.1002/advs.202403387 (PMC11425265; doi:10.1002/advs.202403387)
Supplement: Supplementary file 1 — Supporting Information [file ADVS-11-2403387-s001.docx]

Supporting Information

Intron Retention of DDX39A Driven by SNRPD2 is a Crucial Splicing Axis for Oncogenic MYC/Spliceosome Program in Hepatocellular Carcinoma

*Cunjie Chang#, Lina Li #, Ling Su#, Fan Yang, Quanxiu Zha, Mengqing Sun, Lin Tao, Menglan Wang, Kangli Song, Liangyu Jiang, Haojin Gao, Yexin Liang, Chao Xu, Caiyu Yong, Minmin Wang, Jiacheng Huang, Jing Liu, Weiwei Jin, Wenyuan Lv, Heng Dong, Qian Li, Fangtian Bu, Shuanghong Yan, Haoxiang Qi, Shujuan Zhao, Yingshuang Zhu, Yu Wang, Junping Shi, Yiting Qiao, Jian Xu, Benoit Chabot, Jianxiang Chen**

**Supplementary Figures**

**Figure S1**


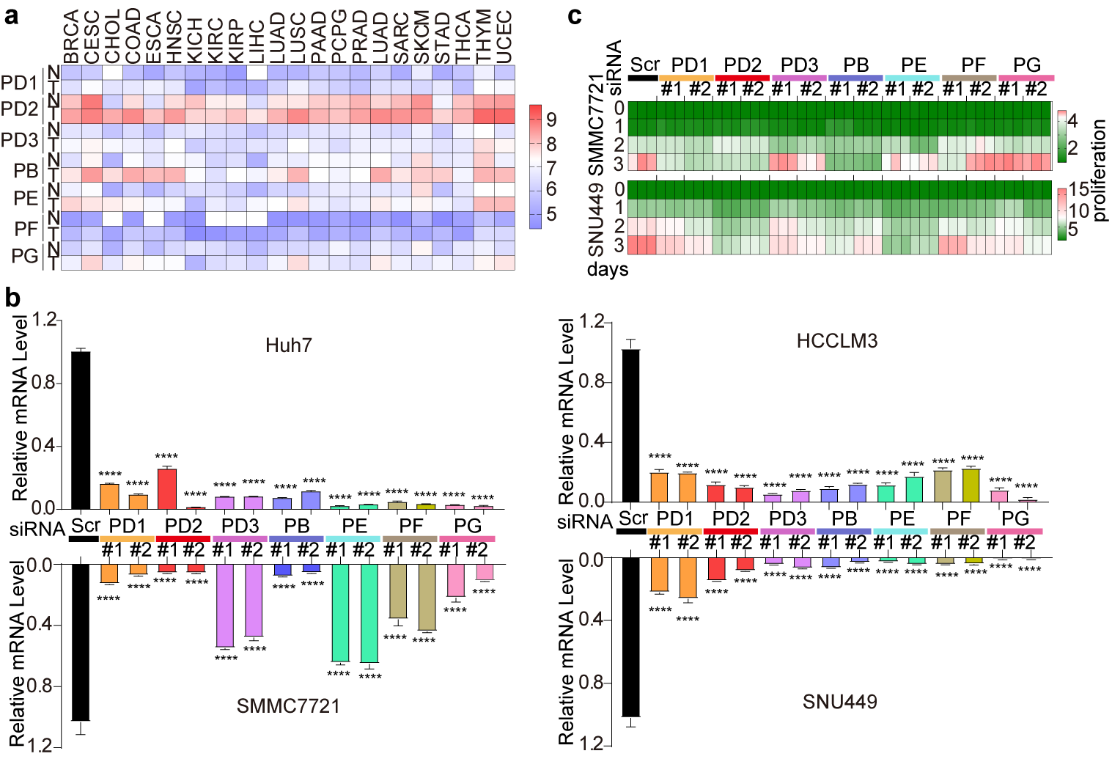


**Figure S1.** PD2 is highly expressed in HCC and negatively correlated with OS. a) The expression of Sm genes in 22 human normal or tumor tissues based on TCGA data. b) qRT-PCR assay was performed to test knockdown efficiency. c) Proliferation index of tumor cells treated with siRNA against Sm proteins in SMMC7721 and SNU449 cells. Two way ANOVA is used for (c), one way ANOVA is used for (b). ^**^p < 0.01; ^***^p < 0.001; ^****^p < 0.0001.

**Figure S2**


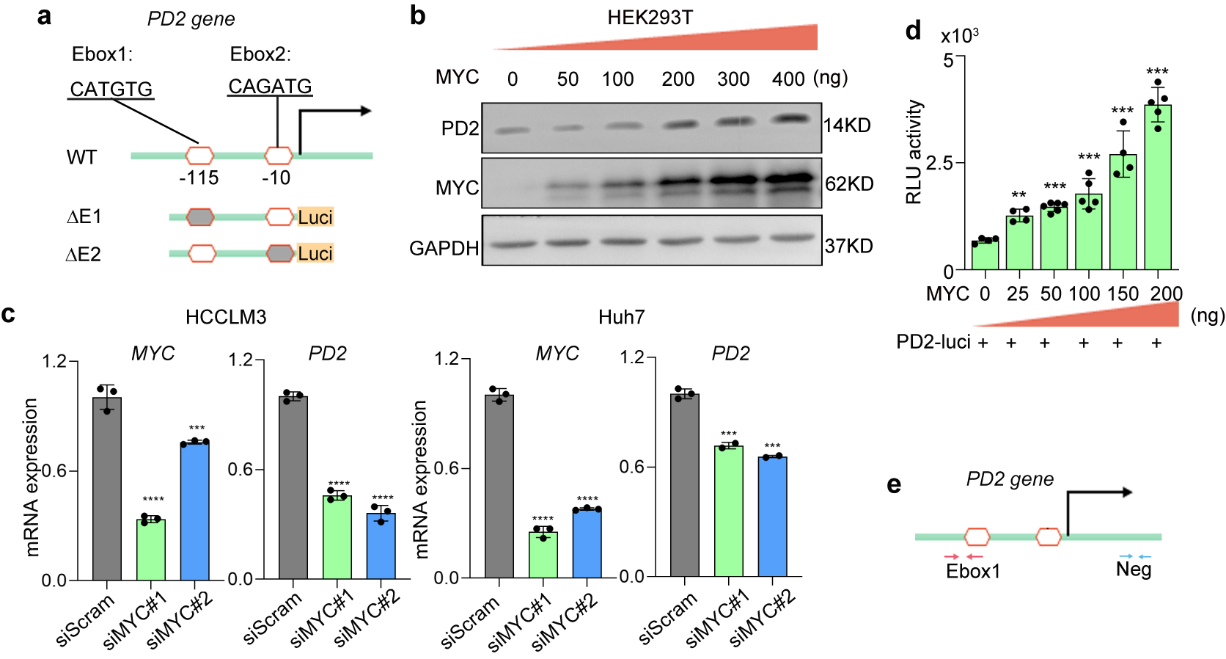


**Figure S2.** MYC directly promotes PD2 transcription through binding Ebox1 element at PD2 promoter. a) Diagram of Ebox1 or Ebox2 in PD2 promoter. Luciferase reporter plasmid (PD2-luci) without Ebox1 or Ebox2 was generated (delta-E1-PD2-luci and delta-E2-PD2-luci). b) WB analysis of PD2 proteins in 293T cells transfected with MYC plasmid in dose-dependent manner. c) The mRNA level of PD2 was detected by qPCR upon MYC knockdown in HCCLM3 and Huh7 cells (n = 3, data are shown as the mean ± SD; ***p <0.001). d) Analysis of luciferase activity of 293T cells transfected with PD2-luci and the indicated MYC-expression plasmid relative to those transfected with control vector. The relative luciferase activity was normalized against the protein concentration of each cell lysate sample. Values mean±s.d. from three independent experiments. e) Primer design for the ChIP experiments. Red primer set localized at Ebox1 region. Blue primer set localized at an intergenic region served as negative control.

**Figure S3**


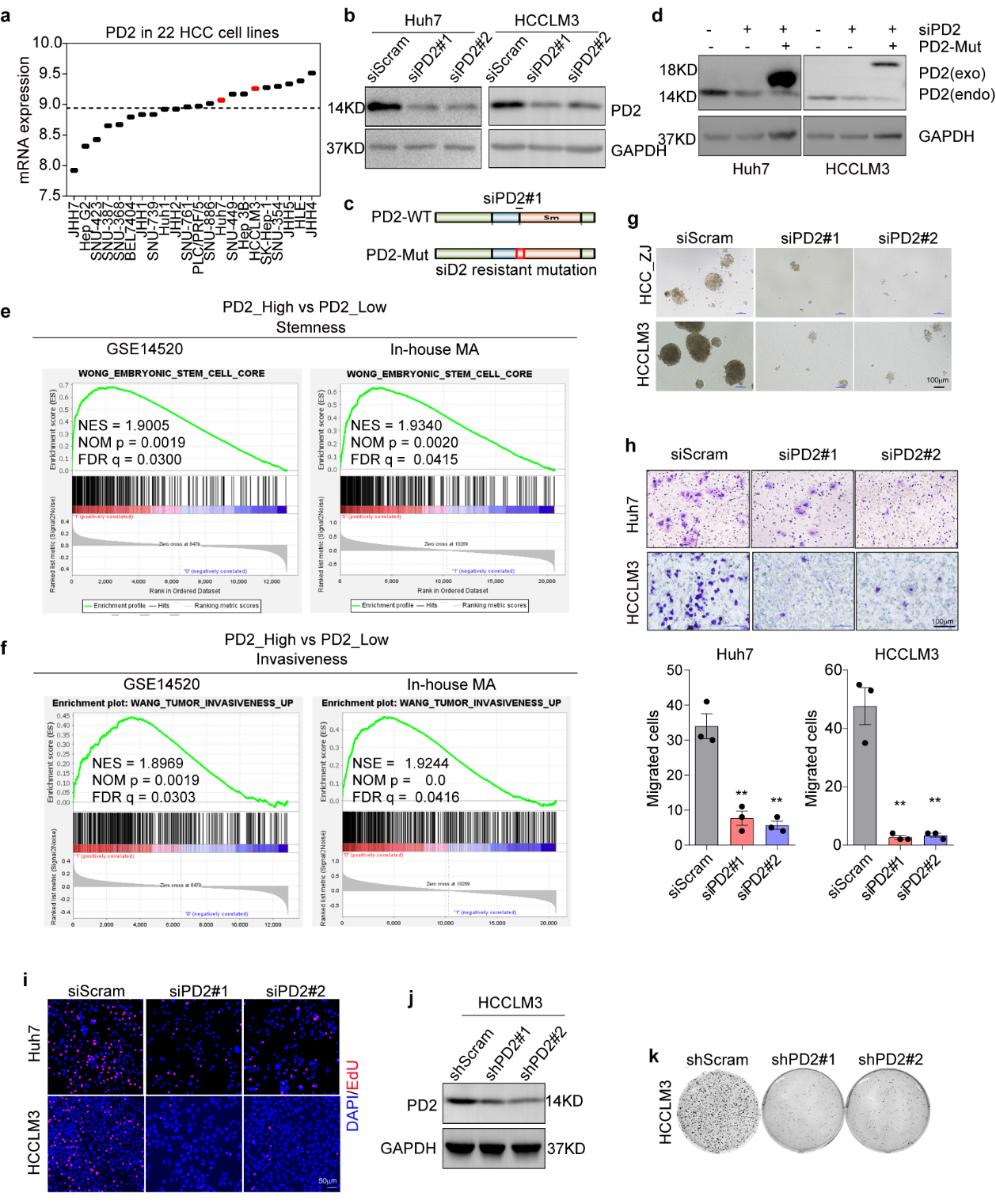


**Figure S3.** PD2 is crucial for HCC progression. a) PD2 expression in 22 HCC cell lines through microarray analysis. b) WB analysis for detection of knockdown efficiency. c) Schematic diagram of siPD2 resistant mutation plasmid. d) WB of endogenous (endo) PD2 and exogenous (exo) PD2-mut (Flag-tagged product of the PD2 synonymous mutant) in the extracts of cells as indicated, loading control: GAPDH. e, f) GSEA analysis of different expressed gene in PD2-high tumor group compared to PD2-low tumor group from GSE14520 dataset. Stemness (e) and invasiveness (f) signature were enriched in PD2-high group. g) Representative area of tumor sphere assay in HCC_ZJ and HCCLM3 cell with or without siRNA against PD2.h) Transwell assay performed with Huh7 and HCCLM3 cells depleted of PD2 by siRNA (#1 and #2) and control non-coding siRNA. Represented images were shown in panel. Migrated cells were quantified. i) Representative images of EdU staining of control and PD2 knockdown Huh7 and HCCLM3 cells. DAPI (blue) was used to stain nucleus and EdU (red) showed the incorporated cells. Scale bar: 50 μm. j) WB analysis of protein harvested from shPD2 and shScram HCCLM3 cells. Each spot represents a biological replicate. Error bars in graphs represent standard deviation. Data were presented as mean ± SEM, *P<0.05; **P<0.01; ***P<0.001. k) Colony formation in soft agar was inhibited by silencing PD2 in stable PD2-knockdown HCCLM3 cells. Represented images were shown.

**Figure S4**


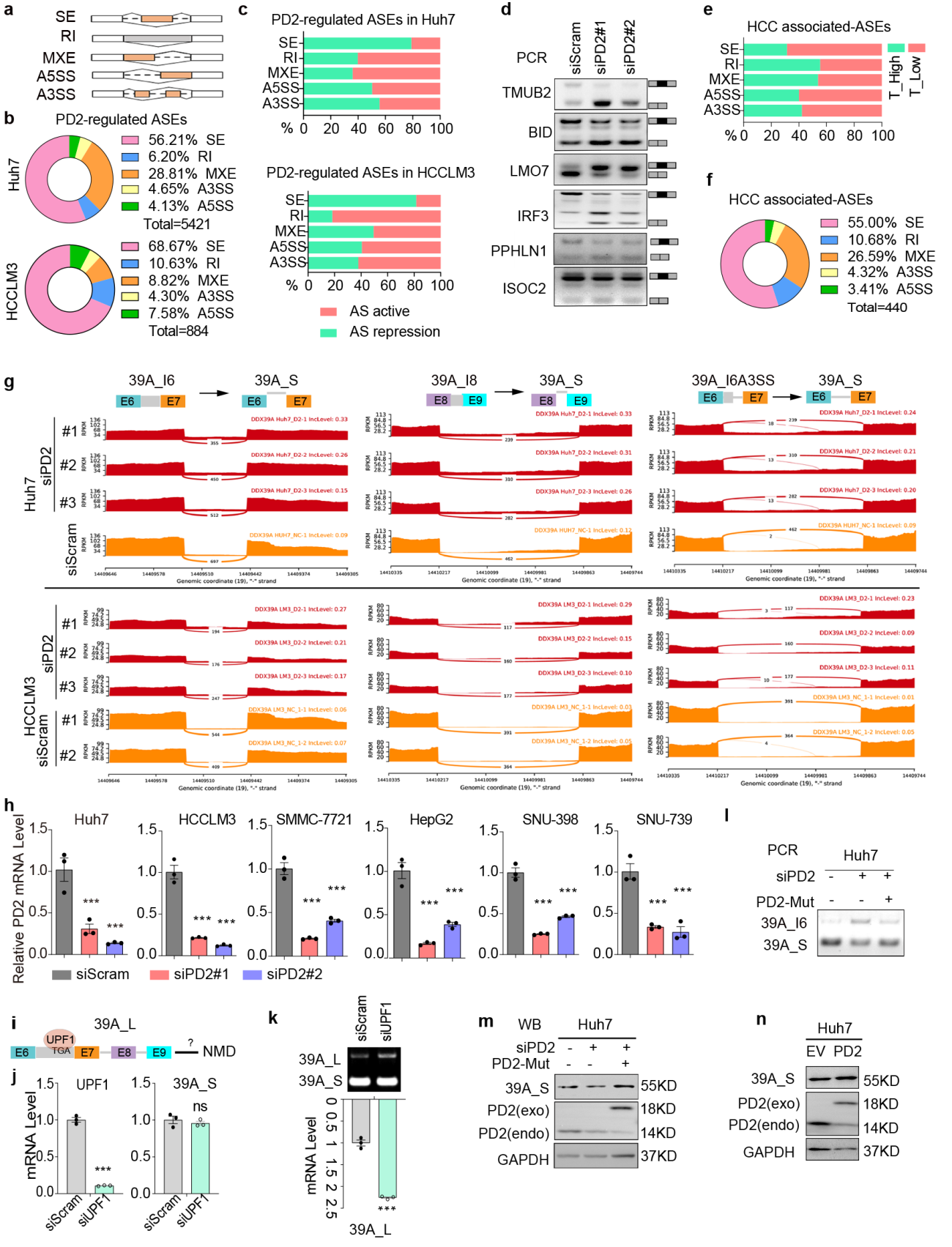


**Figure S4.** PD2 regulates DDX39A splicing. a) The AS events are classified into 5 categories: skipped exon (SE), retained intron (RI), alternative 5′ splice site (A5SS), alternative 3′ splice site (A3SS), and mutually exclusive exon (MXE). b) PD2-regulated AS events in Huh7 and HCCLM3 cell lines. c) Relative fraction of AS events regulated positively (activation) or negatively (repression) by PD2 in each category. d) RT-PCR validation of top six PD2-regulated AS events in Huh7 cell lines. e) HCC related AS events highly spliced genes in human HCC compared matched normal tissues. f) Relative fraction of HCC related AS events in each category. g) Diagram of Reads count of the splicing variants of 39A from RNA_Seq data of cells as indicated. h) PD2 knockdown efficiency in 6 HCC cell lines. i) Diagram of premature stop codon in 39A_I6 transcript. j-k) qPCR (j) and PCR (k) detection of two variants with primer set 1 upon UPF1 silencing in HCCLM3. l) Reintroduction of siRNA-resistant PD2-mut plasmid restored 39A_S expression reduced by PD2 silencing. m) Reintroduction of siRNA-resistant PD2-mut plasmid restored 39A_S protein reduced by PD2 silencing. n) WB detection of 39A_S proteins in PD2 overexpressed Huh7 cells.

**Figure S5**


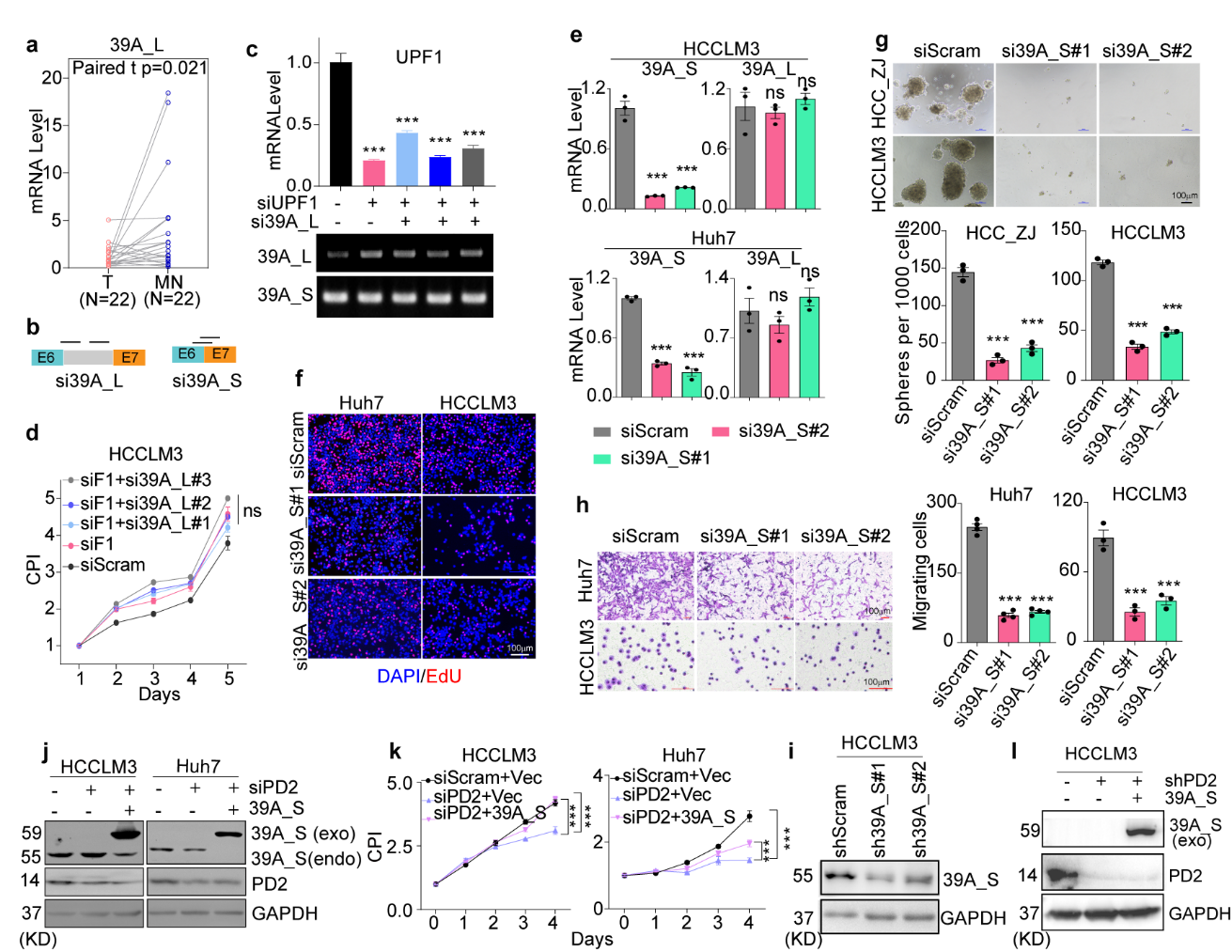


**Figure S5.** 39A_L is a non-coding transcript and has no significant affect on cell proliferation. a) Normalized 39A_L expression ratios in 22 pairs of HCC tissues. b) Diagram of the location of siRNA specifically targeting 39A_S or 39A_L. si39A_S #1 and #2: junction of exon 6 and exon 7-specific siRNAs. c) 39A_L and 39A_S expression and related proliferation index d) in cells co-transfected with siRNA against UPF1 and 39A_L was detected by PCR and qPCR, respectively. e) 39A_L and 39A_S expression detected in two HCC cell lines by qPCR treated with the specificity of siRNA targeting 39A_S (n=3). f) The cell proliferation was analyzed in cells transfected with siScram or si39A_S in two HCC cell lines by EdU incorporation assay (n=3, reprehensive image was shown). g) Representative pictures and quantification of sphere formation in HCC_ZJ and HCCLM3 (n=3). h) Representative image and quantification of migrated cells (n=3). i) WB analysis for knockdown efficiency in 39A_S stably knocked down cells. j) WB detection of 39A proteins and k) relative proliferation index in PD2 silencing cells together with 39A_S overexpression. l) WB analysis of 39A_S protein expression in PD2-knockdown stable cell line infected by lentivirus expressing 39A_S and luciferase or only luciferase. Mean ± SD, two-tailed, unpaired t test is used for (e, g, h), **p < 0.01; ***p < 0.001, ns indicates non-significant. Source data are provided as a Source Data file.

**Figure S6**


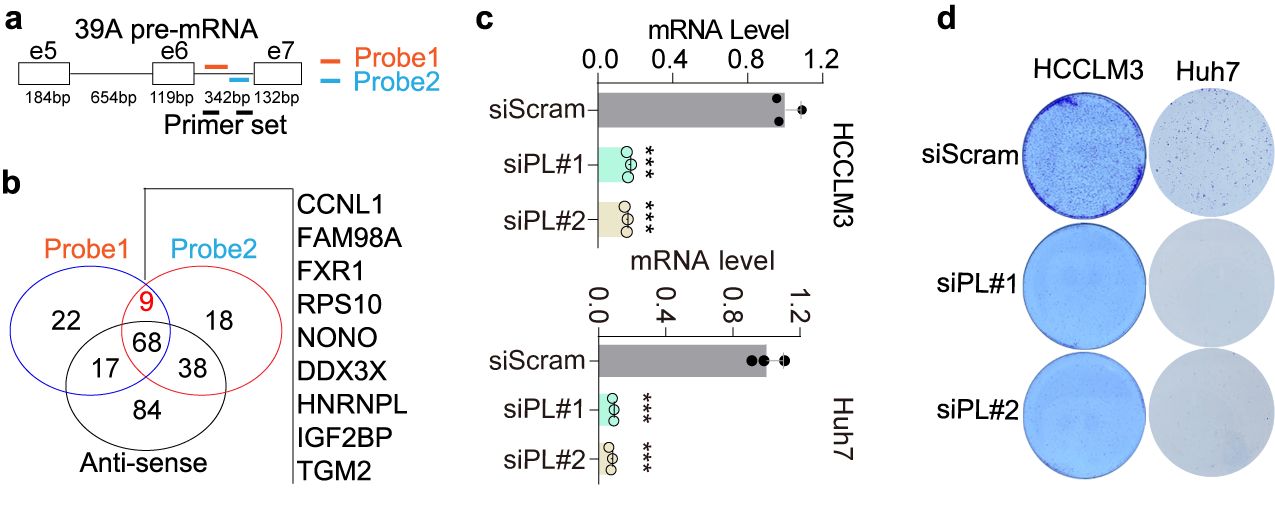


**Figure S6.** PD2 recruits HNRNPL to enhance 39A gene splicing. a) Diagram of the location of probe 1 and 2 for RNA pulldown assay. b) Overlapping of 39A mRNA-associated proteins pulled down by probe 1 and probe 2. c) qPCR detection of HNRNPL knockdown efficiency in HCCLM3 cells (n = 3). d) The 2D-colony formation were analyzed in HCCLM3 and Huh7 cells transfected with siPL. Mean ± SD, two-tailed, unpaired t test is used for (c), **p < 0.01; ***p < 0.001. Source data are provided as a Source Data file.

**Figure S7**


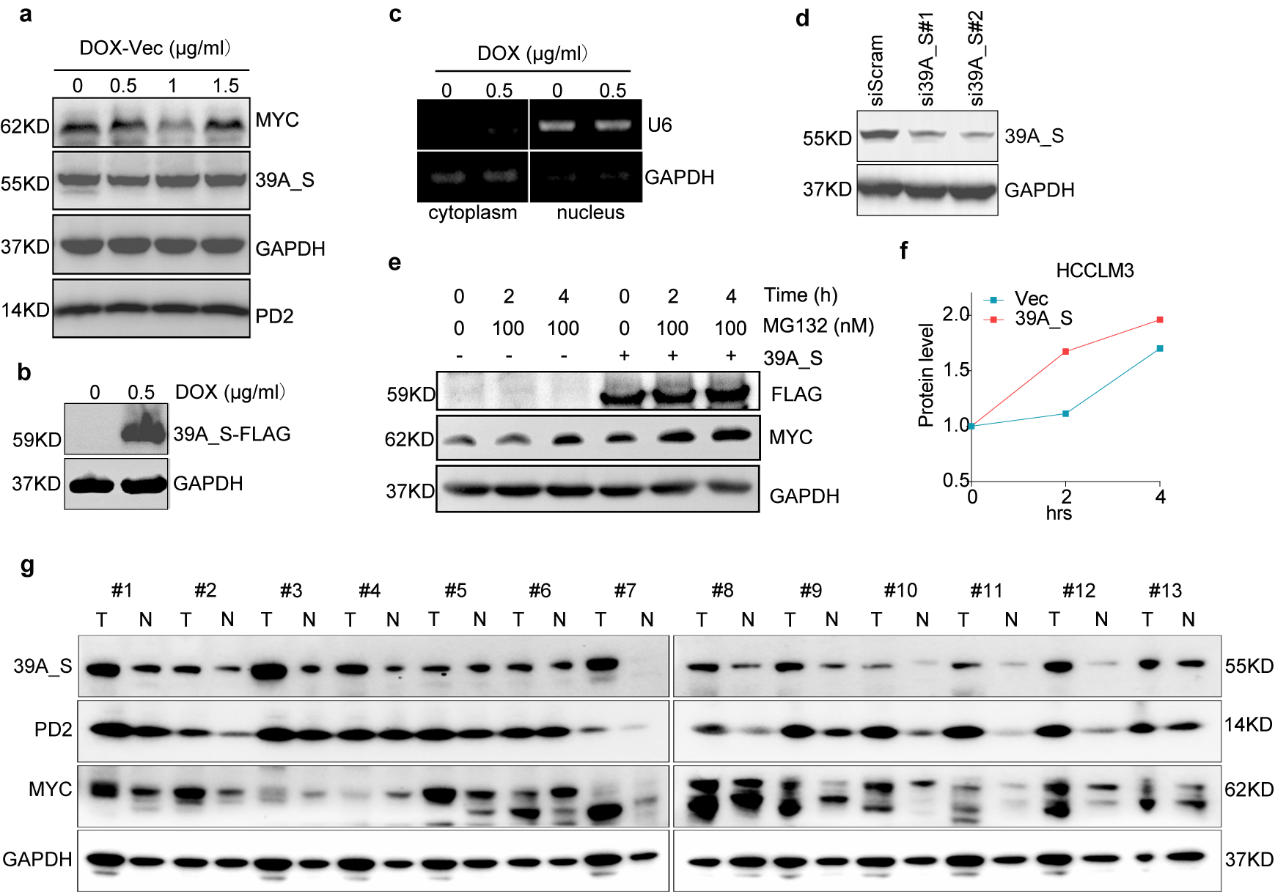


**Figure S7.** MYC/PD2/39A feedback circuits in HCC. a) HCCLM3 cells was infected with Dox-inducible empty vector lentivirus to generate a control cell Dox-Vec stable cell lines. PD2, 39A_S and MYC protein was determined in the control cell treated with doxycycline (DOX). b) HCCLM3 was infected with dox-inducible 39A_S expression lentivirus to generate dox-inducible dox-39A_S stable cell lines, and cell-lysis from dox-39A_S cells treated with or without 0.5 μg/ml doxycycline was detected by WB. c) PCR was performed to test U6 and GAPDH expression in cytoplasm and nuclear component. U6 is a marker for nuclear component and GAPDH is a marker for cytoplasm component. d) WB analysis was used to check the knockdown efficiency of 39A in HCCLM3 cell transfected with siRNA against 39A_S. e, f) Dox-39A_S cells treated with or without doxycycline and MG132 as indicated. The half-life of MYC protein was detected by WB (e) and its qualification (f). g) WB analysis of 13 paired HCC tissues and adjacent normal tissues using antibody against PD2, 39A_S and MYC, respectively.

**Figure S8**


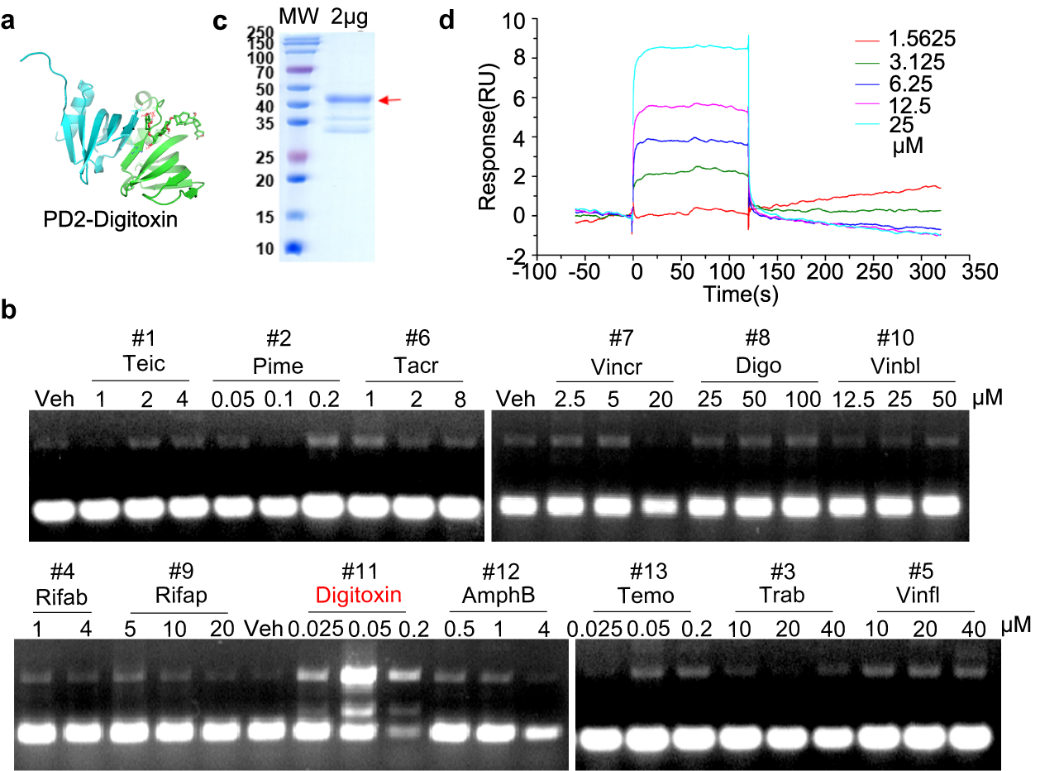


**Figure S8.** Digitoxin inhibits MYC/PD2/39A feedback circuits in HCC. a) Binding region of digitoxin and PD2. b) PCR analysis of 39A_S and 39A_L was performed in HCCLM3 treated with PD2-binding drugs top-listed. Veh: Vehicle, Rifab: Rifabutin, Rifap: Rifapentine, AmphB: Amphotericin B, Temo: Temoporfin, Trab: Trabectedin, Vinfl: Vinflunine, Teic: Teicoplanin, Pime: Pimecrolimus, Tacr: Tecrolimus, Vincr: Vincristine, Digo: Digoxin, Vinbl: Vinblastine. c) Coomassie Brilliant Blue staining checked the expression of human PD2 protein. Red arrow points to the Fc fragment fusion with PD2. d) Representative sensor grams of PD2 interacting with digitoxin.

**Supplementary Tables**

**Table S1 Summary of the clinic pathological characteristics of the 112 HCC patients employed for the IHC and survival analysis in Figure 1f and 1g.**

|  |  |  |  |
| --- | --- | --- | --- |
|  | **PD2-High n=46** | **PD2-Low n=66** | **P Value** |
| **Gender** |  |  |  |
| Male | 33 (71.7) | 57 (86.4) | 0.055 |
| Femal | 13 (28.3) | 9 (13.6) |  |
|  |  |  |  |
| **Age** |  |  |  |
| ≤60y | 33 (71.7) | 39 (59.1) | 0.169 |
| ＞60y | 13 (28.3) | 27 (40.9) |  |
|  |  |  |  |
| **Smoker** |  |  |  |
| No | 26 (56.5) | 31 (47.0) | 0.320 |
| Yes | 20 (43.5) | 35 (53.0) |  |
|  |  |  |  |
| **drink** |  |  |  |
| No | 25 (54.3) | 31 (47.0) | 0.442 |
| Yes | 21 (45.7) | 35 (53.0) |  |
|  |  |  |  |
| **Cancer Embolus** |  |  |  |
| No | 29 (63.0) | 59 (89.4) | **0.001** |
| Yes | 17 (37.0) | 7 (10.6) |  |
|  |  |  |  |
| **Invasion** |  |  |  |
| No | 38 (82.6) | 57 (86.4) | 0.586 |
| Yes | 8 (17.4) | 9 (13.6) |  |
|  |  |  |  |
| **Tumor Size** |  |  |  |
| ≤5 cm | 24 (52.2) | 38 (57.6) | 0.572 |
| ＞5 cm | 22 (47.8) | 28 (42.4) |  |
|  |  |  |  |
|  |  |  |  |
| **Recurrence** |  |  |  |
| No | 12 (26.1) | 41 (62.1) | **<.001** |
| Yes | 34 (73.9) | 25 (37.9) |  |
|  |  |  |  |
| **Survival** |  |  |  |
| alive | 16 (34.8) | 56 (84.8) | **<.001** |
| dead | 30 (65.2) | 10 (15.2) |  |
|  |  |  |  |
| **Hepatitis B e antigen** |  |  |  |
| No | 15 (32.6) | 30 (45.5) | 0.173 |
| Yes | 31 (67.4) | 36 (54.5) |  |
|  |  |  |  |
| **AFP** |  |  |  |
| <=20 | 12 (26.7) | 28 (44.4) | **0.059** |
| >20 | 33 (73.3) | 35 (55.6) |  |
| Missing | 1 | 3 |  |
|  |  |  |  |
| **CEA** |  |  |  |
| <=5 | 41 (95.3) | 61 (98.4) | 0.358 |
| >5 | 2 (4.7) | 1 (1.6) |  |
| Missing | 3 | 4 |  |
|  |  |  |  |
| **CA125** |  |  |  |
| <=35 | 38 (90.5) | 55 (88.7) | 0.774 |
| >35 | 4 (9.5) | 7 (11.3) |  |
| Missing | 4 | 4 |  |
|  |  |  |  |
| **CA199** |  |  |  |
| <=37 | 40 (93.0) | 52 (82.5) | 0.118 |
| >37 | 3 (7.0) | 11 (17.5) |  |
| Missing | 3 | 3 |  |
|  |  |  |  |
| **Tumor No.** |  |  |  |
| 1 | 40 (87.0) | 58 (87.9) | 0.921 |
| >1 | 6(13) | 8(12.1) |  |

**Table S2 Summary of clinic pathological characteristics of the 20 paired HCC patients employed for q-PCR and RNA_Seq.**

|  | **NR n=10** | **R n=10** |
| --- | --- | --- |
| **Gender** |  |  |
| Male | 10 (100) | 8 (80) |
| Femal | 0 (0) | 2 (20) |
|  |  |  |
| **Age** |  |  |
| ≤60y | 8 (80) | 10(100) |
| ＞60y | 2 (20) | 0 (0) |
|  |  |  |
| **Tumor No.** |  |  |
| 1 | 8 (80) | 8 (80) |
| >1 | 2(20) | 2(20) |
|  |  |  |
| **Tumor Size** |  |  |
| ≤5 cm | 7(70) | 5 (50) |
| ＞5 cm | 3 (30) | 5 (50) |
|  |  |  |
| **Survival** |  |  |
| alive | 10 (100) | 4 (40) |
| dead | 0 (0) | 6 (60) |

**Table S3 Summary of the clinic pathological characteristics of the 127 HCC tissues from 76 HCC patients employed for microarray in this study.**

|  | **PD2_Low** | **PD2_High** | **p Value** |
| --- | --- | --- | --- |
|  | **n=44** | **n=32** |  |
| Age |  |  |  |
| <=60 | 20 (45.5) | 16 (50.0) | 0.6952 |
| >60 | 24 (54.5) | 16 (50.0) |  |
|  |  |  |  |
| Cirrhosis |  |  |  |
| Y | 23 (53.5) | 13 (46.4) | 0.5609 |
| N | 20 (46.5) | 15 (53.6) |  |
| missing | 1 | 4 |  |
|  |  |  |  |
| AJCC tumor staging |  |  |  |
| Ⅰ-Ⅱ | 37 (84.1) | 30 (93.8) | 0.1334 |
| Ⅲ | 7 (15.9) | 2 (6.3) |  |
|  |  |  |  |
| Tumor venous infiltration |  |  |  |
| VI | 16 (36.4) | 12 (37.5) | 0.9192 |
| NI | 28 (63.6) | 20 (62.5) |  |
|  |  |  |  |
| Tumor differentiation | |  |  |
| 1 | 6 (13.6) | 3 (9.4) | 0.2458 |
| >=2 | 38 (86.4) | 29 (90.6) |  |
|  |  |  |  |
| Tumor size | |  |  |
| <=5 | 21 (47.7) | 23 (71.9) | 0.0353 |
| >5 | 23 (52.3) | 9 (28.1) |  |
|  |  |  |  |
| AFP serum level | |  |  |
| <=20 | 18 (40.9) | 20 (62.5) | 0.0631 |
| >20 | 26 (59.1) | 12 (37.5) |  |

**Table S4 Primers for plasmid construction.**

| **Primer Name** | **Sequence (5’-3’)** |
| --- | --- |
| pLVX-CMV-SNRPD2-F (PUROMYCIN) | TATTTCCGGTGAATTCATGAGCCTCCTCAACAAGCC |
| pLVX-CMV-SNRPD2-R (PUROMYCIN) | GAGAGGGGCGGGATCCTTACTTGTCATCGTCATCCTTGTAATCGATGTCATGATCTTTATAATCACCGTCATGGTCTTTGTAGTCCTTGCCGGCGATGAGCGGGT |
| pLVX-CMV-DDX39A-201-F (NEOMYCIN) | GGATCTATTTCCGGTGAATTCATGGCAGAACAGGATGTG |
| pLVX-CMV-DDX39A-201-R (NEOMYCIN) | GGAGGGAGAGGGGCGGGATCCTTACTTGTCATCGTCATCCTTGTAATCGATGTCATGATCTTTATAATCACCGTCATGGTCTTTGTAGTCCCGGCTCTGCTCGATGTAT |
| PLVX-CMV-DDX39A-201-F (PUROMYCIN) | GGTCTCGATTCTACG GGATCCATGGCAGAACAGGATGTG |
| PLVX-CMV-DDX39A-201-R (PUROMYCIN) | GATGACGATGACAAGTAATCTAGA CCCAGCTTTCTTGTA |
| PLVX-CMV-SNRPD2-MUT#1-F (PUROMYCIN) | AACACCGGTCCACTCTCTGTTTTAACTCAAAGCGTAAAAAATAATACCCAAGTGCTCATC |
| PLVX-CMV-SNRPD2-MUT#1-R (PUROMYCIN) | GATGAGCACTTGGGTATTATTTTTTACGCTTTGAGTTAAAACAGAGAGTGGACCGGTGTT |
| PLVX-CMV-SNRPD2-MUT#2-F (PUROMYCIN) | AACAATAAGAAACTCCTGGGAAGAGTAAAAGCATTTGACCGACACTGCAACATGGTGCTG |
| PLVX-CMV-SNRPD2-MUT#2-R (PUROMYCIN) | CAGCACCATGTTGCAGTGTCGGTCAAATGCTTTTACTCTTCCCAGGAGTTTCTTATTGTT |
| PLKO-shSNRPD2-1-F  (PUROMYCIN) | CCGGGCTCACACAGTCAGTCAAGAATTCTCGAGAATTCTTGACTGACTGTGTGAGCTTTTTG |
| PLKO-shSNRPD2-1-R  (PUROMYCIN) | AATTCAAAAAGCTCACACAGTCAGTCAAGAATTCTCGAGAATTCTTGACTGACTGTGTGAGC |
| PLKO-shSNRPD2-2-F  (PUROMYCIN) | CCGGGCCGCGUGAAGGCCUUCGAUATTCTCGAGAATATCGAAGGCCTTCACGCGGCTTTTTG |
| PLKO-shSNRPD2-2-R  (PUROMYCIN) | AATTCAAAAAGCCGCGUGAAGGCCUUCGAUATTCTCGAGAATATCGAAGGCCTTCACGCGGC |
| PLKO-shDDX39A-1-F  (PUROMYCIN) | CCGGAAGTTCATGCAGGATCCCATGCTCGAGCATGGGATCCTGCATGAACTTTTTTTG |
| PLKO-shDDX39A-1-R  (PUROMYCIN) | AATTCAAAAAAAGTTCATGCAGGATCCCATGCTCGAGCATGGGATCCTGCATGAACTT |
| PLKO-shDDX39A-2-F  (PUROMYCIN) | CCGGAGTTCATGCAGGATCCCATGGCTCGAGCCATGGGATCCTGCATGAACTTTTTTG |
| PLKO-shDDX39A-2-R  (PUROMYCIN) | AATTCAAAAAAGTTCATGCAGGATCCCATGGCTCGAGCCATGGGATCCTGCATGAACT |
| PCDNA5-minigene-F | CGCGGTGGCGGCCCGGATCCCTGGGTGAGTCCTCTGCCCT |
| PCDNA5-minigene-R | GGCCCCCCCTCGACGAATTCCTGAGGGAAGGAGTGGCAGT |

**Table S5 Primers for real-time PCR.**

| **Name** | **Sequence (5’-3’)** |
| --- | --- |
| SNRPD2-F | GCCGCAACAATAAGAAACTCC |
| SNRPD2-R | TTCCGCAGGACCACGATG |
| DDX39A_S-F | AGTTCATGCAGGATCCCAT |
| DDX39A_S-R | TCCAGCACATCCAAGAGATCA |
| DDX39A_L-F | AGCGATGTTTACTGAGGTC |
| DDX39A_L-R | AACTCCAGCACATCCAAGA |
| HNRNPL-F | AACTACGCAGCCGACAAC |
| HNRNPL-R | CATCGCCTGAACTCCATT |
| SNRPD1-F | GCTTCCGGCCATTCATAC |
| SNRPD1-R | GAATCCTTCAACCGAACACT |
| SNRPD3-F | TTCGCCGTAGCATCTTTCG |
| SNRPD3-R | CTGGCAGTTCATGTTGTCCTCT |
| SNRPB-F | TTGGCACCTTCAAGGCTTTTGAC |
| SNRPB-R | AGACCGAGGACTCGCTTCTCTT |
| SNRPE-F | AAGTGAATATGCGGATAGA |
| SNRPE-R | CCAATGATACAGCCTTCT |
| SNRPF-F | CTGCTGTAGTCACGAGGGA |
| SNRPF-R | AGGAAAGGTTTGGGATTG |
| SNRPG-F | TCCCTTTATGAACCTTGT |
| SNRPG-R | TGATGATACTATTTCCTCGT |
| MYC-F | TCAAGAGGCGAACACACAAC |
| MYC-R | GGCCTTTTCATTGTTTTCCA |
| UPF1-F | ACCGACTTTACTCTTCCTAGCC |
| UPF1-R | AGGTCCTTCGTGTAATAGGTGTC |
| Probe1-F | TAGACATGCGGCGGGATGT |
| Probe1-R | CTACTCACATCCTGCATGAACT |
| Probe2-F | TACTGAGGTCTGGTGCAGCA |
| Probe2-R | GTCGTCCACAAACACCTCCAT |
| CHIP-D2-△1-Q-F | CTCCTCAGGCAGCAGCAAC |
| CHIP-D2-△1-Q-R | ACACTCAATCGGTCAAATATCCA |
| RIP-PL-39A-F-Q | TGAGCGATGTTTACTGAGGTCTG |
| RIP-PL-39A-R-Q | TGCGGTTCTTCTCACTGTCTTT |
| GAPDH-F | ATGGGGAAGGTGAAGGTCG |
| GAPDH-R | GGGGTCATTGATGGCAACAATA |
| DDX39A-F | AGTTCATGCAGGATCCCAT |
| DDX39A-R | TCCAGCACATCCAAGAGATCA |
| PPHLN1-F | TTACAGAAGAGACGAAATG |
| PPHLN1-R | CTTGTCCATCTATAACCA |
| BID-F | GCCATAAGGAGGAAGCGGGTAG |
| BID-R | GGGCGAGGTGCCTGGCAATA |
| TMUB2-F | GGGTTAGTTGTGGTTGTGATAAA |
| TMUB2-R | GGTTGCTACCGCTGTCTGCT |
| IRF3-F | CTCTGAGAACCCACTGAAGCG |
| IRF3-R | CCTTGTACTGGTCGGAGGTGA |
| LMO7-F | AAGGATTTCGGCTGTTGAG |
| LMO7-R | TTCTGCGCTGACATCACTC |
| ISOC2-F | AGTTCCGCCACAACATCGC |
| ISOC2-R | CGGTCTAGGAGGTCCAGGGT |

**Table S6 siRNA sequences.**

| **siRNA sequence** | | |
| --- | --- | --- |
| **Primer Name** | **sense (5’-3’)** | **antisense (5’-3’) (dTdT)** |
| siSNRPD2-1 | GCUCACACAGUCAGUCAAGAATT | UUCUUGACUGACUGUGUGAGC |
| siSNRPD2-2 | GCCGCGUGAAGGCCUUCGAUATT | UAUCGAAGGCCUUCACGCGGC |
| siDDX39A_S-1 | AAGUUCAUGCAGGAUCCCAUG | CAUGGGAUCCUGCAUGAACUU |
| siDDX39A_S-2 | AGUUCAUGCAGGAUCCCAUGG | CCAUGGGAUCCUGCAUGAACU |
| siUPF1-1 | GAUGCAGUUCCGCUCCAUU | AAUGGAGCGGAACUGCAUC |
| siDDX39A_L-1 | AUGUUUACUGAGGUCUGGUGC | GCACCAGACCUCAGUAAACAU |
| siDDX39A_L-2 | GCCAGAGCUUACAUCCAGCUU | AAGCUGGAUGUAAGCUCUGGC |
| siDDX39A_L-3 | UGAGUCGGGCCAGAGCUUACA | UGUAAGCUCUGGCCCGACUCA |
| siMYC-1 | GGAAACGACGAGAACAGUU | AACUGUUCUCGUCGUUUCC |
| siMYC-2 | GGUCAGAGUCUGGAUCACC | GGUGAUCCAGACUCUGACC |
| siDDX39A-total-1 | CCCUACAGCAGAUUGAGCCUGUCAA | UUGACAGGCUCAAUCUGCUGUAGGG |
| siDDX39A-total-2 | GUUGAGAAGCUAGAGAUUGUAUGAG | CUCAUACAAUCUCUAGCUUCUCAAC |
| siHNRNPL-1 | CAUCAUGCCUGGUCAGUCA | UGACUGACCAGGCAUGAUG |
| siHNRNPL-2 | AUAGUGUAAAGAACAUCCG | CGGAUGUUCUUUACACUAU |
| siSNRPD1-1 | GCUGCAUAGUUGAAGAUUUAU | AUAAAUCUUCAACUAUGCAGC |
| siSNRPD1-2 | CCUAAGGUGAAAUCUAAGAAA | UUUCUUAGAUUUCACCUUAGG |
| siSNRPD3-1 | AGUGGCACAGCUGGAGCAGGU | ACCUGCUCCAGCUGUGCCACU |
| siSNRPD3-2 | GAAGAACGCACCCAUGUUA | UAACAUGGGUGCGUUCUUC |
| siSNRPB-1 | CCACAAGGAAGAGGUACUGUU | AACAGUACCUCUUCCUUGUGG |
| siSNRPB-2 | CACAUGAAUUUGAUCCUCUGU | ACAGAGGAUCAAAUUCAUGUG |
| siSNRPE-1 | CCCUCGUGUUACUACAAGATT | UCUUGUAGUAACACGAGGGTA |
| siSNRPE-2 | GCUCUAUGAGCAAGUGAAU | AUUCACUUGCUCAUAGAGC |
| siSNRPF-1 | AUGAACAUGCAGCUUGCAA | UUGCAAGCUGCAUGUUCAU |
| siSNRPF-2 | UAUCAGAGGUGUGGAAGAA | UUCUUCCACACCUCUGAUA |
| siSNRPG-1 | AUACGAGGAAAUAGUAUCA | UGAUACUAUUUCCUCGUAU |
| siSNRPG-2 | GGAUUUGAUCCCUUUAUGA | UCAUAAAGGGAUCAAAUCC |

**Table S7 Probe sequences.**

| **Probe sequence** | | |
| --- | --- | --- |
| **Name** | **Sequence** | **Modifications** |
| MYC-probe（E2,E3-J） | TTCTTCCTCATCTTCTTGTTCCTCCTCAGAGTCGCTGCTGGTGGTGG | 5‘alexa594；Texas red |
| DDX39A-6I-1 | GAGGUCUGGUGCAGCAGGUCCUCUGGGAAGUGUCGCACA |  |
| DDX39A-6I-2 | AUGCGGCGGGAUGUGCAGGAGAUCUUCCGCCUGACACCA |  |
| DDX39A-antisense | AGAGGUGAUCCCUGCCCGCCCCUCCCACCUCCCUUCCCA |  |

**Table S8 List of the antibodies used in the study.**

| **Antibodies used for Western blot** | | | | | |
| --- | --- | --- | --- | --- | --- |
| **Cat.No** | **Antibody** | | **Species** | **Dilution** | **Company and description** |
| 2118S | GAPDH | | Rabbit | 1/5000 | Cell Signaling Technology |
| 9402S | MYC | | Rabbit | 1/1000 | Cell Signaling Technology |
| ab198296 | SNRPD2 | | Rabbit | 1/2000 | Abcam |
| PA5-31220 | DDX39A | | Rabbit | 1/1000 | Thermofisher |
| sc32317 | hnRNPL | | Mouse | 1/1000 | Santa Cruz Biotechnology |
| A8592 | Flag | | Mouse | 1/5000 | Thermofisher |
| sc40 | MYC | | Mouse | 1/1000 | Santa Cruz Biotechnology |
| 11723-1-AP | DDX39A | | Rabbit | 1/1000 | Proteintech |
| **Antibodies used for IHC and ChIP** | | | | | |
| **Cat.No** | | **Antibody** | **Species** | **Dilution** | **Company and description** |
| SAB5300423 | | Ki67 | Mouse | 1/200 | Sigma, IHC |
| ab198296 | | SNRPD2 | Rabbit | 1/500 | Abcam, IHC |
| 11723-1-AP | | DDX39A | Rabbit | 1/50 | Proteintech, IHC |
| sc40 | | MYC | Mouse | 1/50 | Santa Cruz Biotechnology, IHC |
| 9402S | | MYC | Rabbit |  | Cell Signaling Technology, ChIP |

**Meterials and methods**

*Cell growth, proliferation, survival, and migration assays*: The growth of cells was quantified with CCK-8 (Meilunbio, Cat. # MA0218), and the proliferating cells were labeled by 5-ethynyl-2’-deoxyuridine (EdU) staining (RIBOBIO, Cat. #C10310-3). The ability of anchorage-dependent growth was assessed by colony formation assays and the ability of anchorage-independent proliferation was assessed by soft agar assay. Cell migration assay was determined by the number of migration cells to the lower side of the transwell filters (Costar, Corning Incorporated).

*Dual-luciferase reporter assay*: For dual-luciferase report assay, we constructed three sets of *PD2* promoter luciferase reporter plasmids accordingly. The wild-type *PD2* promoter sequence containing two predicted E-box sites (Ebox1 and Ebox2) was cloned in the pGL3-basic luciferase reporter vector (PD2-luci). Mutant *PD2* promoter sequence without Ebox1 site or Ebox2 site was constructed into the same vector (∆E1-luci and ∆E2-luci). HEK293T cells were co-transfected with *PD2* promoter plasmid vector (wildtype or mutant) and MYC over-expression vector and pRL Renilla luciferase plasmid (Promega, Cat. #E2231) with PolyJet (SignaGen, Cat. #SL100688). After 24 h, Luciferase activity was measured by the dual-luciferase reporter assay system (Promega, Madison, WI, USA, Cat. #E2920) according to the manufacturers protocol. Firefly luciferase was normalized to Renilla luciferase activity. Experiments were performed at least three biological repeats. The primers used for promoter luciferase plasmids constructs are listed in the Table S4.

*siRNA*: The siRNAs targeting genes were purchased from Gene Pharma and transfected with Lipofectamine RNAiMAX (Thermo Fisher Scientific, 13778150). The sequences are listed in Table S6.

*Western blotting and Immunoprecipitation (IP)*: Western blotting cells were cultured in 6-well plates, and treated with siRNA. After 72 h, cells were collected in 1.5mL tubes and lysed with RIPA lysis buffer (Thermo, the United States, Cat. # TG268762) containing protease inhibitor cocktail (Sigma-Aldrich, the United States, Cat. #27423500) and DTT on ice. After removal of insoluble debris by centrifugation maximum speed, the supernatant was boiled with loading buffer containing Sodium Dodecyl Sulfate (SDS) to denature proteins. For IP, pellet was resuspended in IP lysis buffer (20 mM Tris pH 8, 10% glycerol, 150 mM NaCl, 0.1% NP-40, 0.1 mM EDTA) and sonicated using Bioruptorplus Plus (UCD-300) 5 sec pulses with 5 sec rest between each pulse (5 cycles). The lysate was pelleted by spinning at 13000 g for 20 min. Pre-clear the lysate by incubating with magnetic beads at 4 ℃ with gentle rotation for 45 min. Then transfer the precleared supernatant into a new tube after placing the tubes on a magnetic rack for 2 min. To pulldown the IP proteins, add appropriate M2 Gel (Sigma, Cat. # A2220) and incubate in 4 ℃ for 4 h with gentle rotation. After that, the M2 Gel was collected by spinning at 4500g for 5 min. Discard the supernatant. Then wash the M2 Gel using low salt IP lysis buffer (150 mM NaCl) for 5 times, high salt IP lysis buffer (500 mM NaCl) for 1 time. Discard the supernatant. Add 2×SDS loading buffer to the M2 Gel. Boiled the samples at 100 ℃ for 10 min. Discard the M2 Gel following spinning at 4500 g for 1 min. Then, the proteins were separated by SDS-PAGE and transferred onto polyvinylidene fluoride (PVDF) membranes (Merck Millipore, the United States, Cat. #IPVH00010). The membranes were blocked with 5% nonfat dry milk (BioFROXX, Germany, Cat. #1172GR500) for 1 h, and incubated with primary antibodies overnight at 4℃. The next day, they were incubated with specific anti-mouse (Abcam, ab99697) or anti-rabbit (Abcam, ab21234) second antibodies for 1 h, and detected protein levels by immunoblot. The antibodies information in detail is summarized in Table S8.

*Plasmids and lentiviral transduction*: Primers used for plasmids constructs were listed in the Table S4. To ectopically express PD2, 39A_S or MYC, the PD2, 39A_S and MYC cDNA constructs were transfected in target cells using Polyjet (SignaGen, Cat. #SL100688) according to the manufacturer’s instruction. Cells were collected for further analysis after 48 h. To establish stably silenced gene cell lines, shRNA oligomers were designed, annealed, and inserted into Lentiviral cloning vectors pLKO.1 GFP shRNA (Addgene Cat. # 8453) according to Addgene pLKO.1 protocol. The sequences of shRNA were showed in the Table S4. Lentiviral were produced in 293FT cells by co-transfecting with the pLKO.1 vectors, an envelope plasmid and two packing plasmids. 48 h after transfection, the supernatant containing virus particles was collected to transduce HCCLM3 and Huh7 cells in the presence of 5 μg/mL polybrene (SantaCruz, Cat. # sc-134220). The infected cells were selected for puromycin (Beyotime, Cat. # ST551) or blasticidin (Invivogen, Cat. # ant-bl-1) resistance for one week. Knockdown efficiency was detected by QPCR and WB analysis.

*RNA isolation, Reverse-transcriptase PCR (RT-PCR), quantitative reverse transcriptase PCR (qRT-PCR)*: The total RNA of cells was isolated using the TRIzol reagent (Invitrogen, Cat. # 15596026) following the standard protocol. The concentrations of RNA were determined by Nanodrop One (Thermo Fisher Scientific Inc., MA, USA). The cDNAs were prepared by HiScript III RT SuperMix for qPCR (Vazyme, Cat. # R323). qRT-PCR of the mRNAs under study was performed using the ChamQ Universal SYBR qPCR kit (Vazyme, Cat. # Q711-02). The specific primers were synthesized by Zhejiang Sunya Biotechnology Co., Ltd, whose sequences are listed in Table S5. The normalized and fold changes of the mRNA levels were identical to the 2^–ΔΔCt^ method. To detect the isoforms of *PD2*-guided AS, PCR was performed using the 2 × Taq Master Mix (Vazyme, Cat. # P111-02) following manufacturers instruction.

*Nuclear and cytoplasmic RNA extraction*: For detection of the nuclear and cytoplasmic MYC, HCCLM3 cells were transfected by *pLVX-TetOne-DDX39A_S* plasmid. 1 × 10^6^ cells in 6 cm dish were collected and washed with 1 mL ice-cold PBS by pipetting for two more times. Resuspend the cell pellet in 350 μL ice-cold hypotonic buffer (10 mM Tris-HCl, pH 7.9, 1.5 mM MgCl_2_, 10 mM KCl, 1 mM DTT and 1× RNase inhibitor) by pipetting for 30 min on ice. Centrifuge the lysate at 2000 rpm for 10 min at 4 ℃. Carefully transfer the supernatant into a fresh microcentrifuge tube. Store on ice. This fraction primarily contains cytosolic RNA. Wash the pellet in 1 mL ice-cold hypotonic buffer by pipetting. Centrifuge the suspension at 2000 rpm for 10 min at 4 ℃. Repeat four more times. Add 500 μL TRIzol reagent to cytosolic supernatant and nuclear pellet. Use RNA isolation (Sangon Biotech, Cat. # B615008), qRT-PCR method for further RNA analysis.

*RNA pulldown*: Affinity purification of DDX39A exon6-exon7 interacting splicing proteins was performed in HCCLM3 cells using two positive probes binding to DDX39A pre-mRNA and one negative control probe (Zhejiang Sunya Biotechnology Co., Ltd). Cells were UV-crosslinked with 150 mJ/cm^2^ (UVP Crosslinker CL-1000, Analytik Jena AG), harvested in ice-cold PBS and lysed in Polysome buffer buffer (pH 7.5, 25mM Tris-HCl, 150mM KCl, 0.5mM DTT, 0.5% NP-40 with RNase Inhibitor, protease inhibitor cocktail). Samples were sonicated5 sec pulse with 5 sec rest between each pulse (5 cycles) (Bioruptorplus Plus (UCD-300) and the protein concentration of the supernatant after centrifugation for 20 min at 12,000 rpm at 4 ℃ was quantified to 2 mg/mL, and we prepared probe magnetic beads by co-incubated the magnetic beads (Thermo Fisher, Cat. 65501) and denatured probes in RNA capture buffer (pH 7.5, 20mM Tris-HCl, 1M NaCl, and 1mM EDTA + RNase Inhibitor) for 30 min at room temperature. Then we divide the cell lysate into three parts and take 10% as input. The prepared beads were washed three times with NT2 buffer (pH 7.4, 50mM Tris-HCl, 150 mM NaCl, 1mM MgCl2, 0.05% NP-40) to elute the non-specific binding. Protein-RNA pulldowns were performed for 4 h at 4℃ with gentle rotation with prepared probe magnetic beads. Affinity purification were washed five times in ice-cold low salt NT2 buffer (150 mM NaCl) and one times in high salt NT2 buffer (500 mM NaCl). Proteins eluted in SDS-PAGE loading buffer for 10 min at 100 ℃. Samples were analyzed by MS.

*RNA Binding Protein Immunoprecipitation (RIP)*: RIP was performed in HCCLM3 cells transfected with pLVX-CMV-VECTOR and pLVX-CMV-*PD2* plasmids. 3 × 10^7^ cells were fixed in 0.1% formaldehyde for 10 min at room temperature and quenched with 250 mM glycine for 5min at room temperature. Then cells were harvested with cold PBS and resuspended in RIP lysis buffer (RIP lysis buffer contained protease inhibitor cocktail, RNase inhibitor and 0.5mM DTT). The magnetic beads were coated with anti-hnRNPL (Santa Cruz, Cat. #sc32317) to prepare them for immunoprecipitation. The cell lysate was centrifuged at 12,000 rpm for 20 min at 4 ℃. Prewashed protein A / G (Protein A, Thermo Fisher Scientific, Cat. #91219463; Protein G, Thermo Fisher Scientific, Cat. #01134323) was added to the cell lysate and pre-blocked at 4 ℃ for 30 min. Next, protein A / G was removed and pre-blocked cell lysate was added to tubes containing the beads-antibody complex in a RIP immunoprecipitation buffer (860 µL RIP wash buffer, 35 µL 0.5 M EDTA, 5 µL RNase inhibitor). Tubes were incubated under rotation at 4 ℃ for 3 h. Then, tubes were placed on a magnetic separator, and the supernatant was discarded. The beads were washed six times. Each bead and input samples were then resuspended in decrosslinking buffer (contain 3% proteinase K, 15mM DTT and RNase inhibitor), and all tubes were incubated at 42 ℃ for 1 h and then 55 ℃ for 1 h to digest the protein. After proteinase digestion, add TRizol reagent to tubes and extract RNA by phenol-chloroform-isoamylalcohol and ethanol precipitation. Last, we used qRT-PCR assay to detect DDX39A transcript using the following primers: RIP-PL-39A-F-Q: TGAGCGATGTTTACTGAGGTCTG, R-Q: TGCGGTTCTTCTCACTGTCTTT

*Chromatin Immunoprecipitation (ChIP)*: HEK293T cells were transfected with PLVX-CMV-MYC plasmid and grown in 10 cm dish to 80% confluence for each ChIP reaction. Then ChIP was done using the Chromatin Immunoprecipitation (ChIP) Kit (BersinBio, Cat. # Bes5001) and according to the protocol provided. Antibodies used were anti-MYC (CST, Cat. #9402S). Briefly, cells were fixed in 1% formaldehyde for 10 min at 37 ℃ and quenched with 1.375 M glycine for 5 min at 37 ℃. Cells were subsequently washed with cold PBS. Nuclei were extracted in Lysis buffer and chromatin was sonicated to 200-600bp. Sonicated chromatin was used in immunoprecipitation reactions with indicated antibodies and IgG antibodies overnight followed by capture using Protein G Dynabeads for 30 min. Captured antibody-chromatin complexes were washed, eluted, and treated with RNase A for 1 h at 37 ℃ and Proteinase K for 2 h at 55 ℃. ChIP DNA was extracted using AxyPrep PCR cleanup Kit (Axygen, Cat.#AP-PCR-250) and enrichment was detected by qRT-PCR analysis. The primers and antibody were listed in Table S5 and S8, respectively.

*Immunohistochemistry (IHC) analysis*: Bake the slides in a 55 °C oven for 4 h so that the tissues are firmly attached to the slides. Then, slides were deparaffinized in xylene 2 times for 10 min each and rehydrated with a series of graded alcohol (50%, 70%, 80%, 90%,100%) for 2 min. The closed endogenous peroxidase was incubated in 3% hydrogen peroxide (prepared with methanol). Antigen retrieval was performed in steamer by heat the slides in citrate buffer (pH 6; Sangon Biotech, Cat. #E673002). Then the slides were incubated with primary antibody in a humidified container at 4°C overnight after blocked with 5% Bovine serum albumin (BSA) for 30 min (The concentration of the antibodies used is shown in the Table S8). The next day, the slides were rinsed in PBS with gentle agitation and incubated with secondary antibody diluted to 1:1000 with 1% BSA for 1 h at room temperature. Immunohistochemical staining was performed with DAB kit (Sangon Biotech, Cat. #E670033) according to the manufacturer’s instructions. Nuclei staining was performed using Hematoxylin staining solution (Sangon Biotech, Cat. #E607317) for 15-20 sec and then drop the slides in 0.1% HCl for 15-20 sec. Slides were subsequently dehydrated in graded alcohol and sealed with Neutral Balsam Mounting Medium (Sangon Biotech, Cat. #E675007). The grade of staining intensity was evaluated on a scale of 1 to 10 according to the percentage of positive cells. Scored independently by three pathologists, mean IHC score was used to stratify high- and low-expression group.

*Immunofluorescence staining*: Cells were fixed with 4% paraformaldehyde (PFA, Absin, Cat. #Abs9179), permeabilized with 0.4% Triton X-100 (Sangon, Cat. #9002-93-1). Rabbit anti-human PD2 antibody was purchased from Abcam (Cat. #ab198296). Rabbit anti-human DDX39A antibody was purchased from thermo (Cat. #PA5-31220). Chicken anti-human Tubulin was used to stain cell cytoplasm. Alexa Fluor® 594 goat anti-rabbit (Molecular Probes, A-11037), Alexa Fluor® 488 goat anti-mouse (Molecular Probes, A-11029) or Alexa Fluor® 633 goat anti-chicken (Molecular Probes, A-21103) was used to label target proteins and DAPI reagent-stained cell nuclei. Treated cells were detected using an FV-1000 laser-scanning confocal microscope (Olympus).

*RNA-fluorescence in situ hybridization (FISH)*: Experimental operations were performed in a RNase-free environment. Cells on coverslips in a 12-well plate were transfected with siRNA and cultured for 36 to 48 h, fixed with 4% PFA in PBS for 15 min, and permeabilized with 0.5% Triton X-100 for 10 min. Cells were washed with graded alcohol (70%, 90%, 100%) for 3 min each time. Then Alexa Fluor® 594-labeled MYC probes (Zhejiang Sunya Biotechnology Co., Ltd) were diluted to 1 μM and Alexa Fluor® 594-labeled poly (A) probes (Invitrogen) were diluted to 10 pM in hybridization buffer. Probes were denatured for 5 min at 88℃ and incubated overnight (12-18 h) with cells in a humidified chamber at 37℃. The next day, cells were washed three times with PBS for 5 min each time, and once incubated with DAPI reagent for 15 min and F-actin reagent for 30 min. Fluorescent images were captured at random using an FV-1000 laser-scanning confocal microscope (Olympus). Sequence of MYC-probes were listed in Table S7.

*Minigene report assay*: Using genomic DNA from HCCLM3 as a template, we amplified the fragment spanning from intron 5 to intron 7 of 39A and inserted this fragment into the pCDNA5 plasmid to generate the minigene reporter. The minigene reporter and PD2-expressing plasmid were transfected into 293T cells. 24 hours later, RNA was extracted, and the short and long splicing fragments were detected using primers localized at the FN1 intron element of the plasmid by RT-PCR and PCR.
